# Changes in Phenolic Metabolites and Biological Activities of Pumpkin Leaves (*Cucurbita moschata* Duchesne ex Poir.) During Blanching

**DOI:** 10.3389/fnut.2021.641939

**Published:** 2021-03-15

**Authors:** Florence M. Mashitoa, Tinotenda Shoko, Jerry L. Shai, Retha M. Slabbert, Dharini Sivakumar

**Affiliations:** ^1^Phytochemical Food Network Research Group, Department of Crop Sciences, Tshwane University of Technology, Pretoria, South Africa; ^2^Department of Horticulture, Tshwane University of Technology, Pretoria, South Africa; ^3^Department of Biomedical Sciences, Tshwane University of Technology, Pretoria, South Africa

**Keywords:** leafy vegetable, phytochemicals, antioxidant activity, post-harvest processing, bioactivity

## Abstract

Pumpkin leaves (*Cucurbita moschata* Duchesne ex Poir.) are popularly consumed in Sub-Saharan Africa and Asia. Blanching the leaves before drying is a method of preservation during off-season. In this study, different blanching treatments and media are used to test the changes in non-targeted phenolic compounds, antioxidant capacity (FRAP and ABTS activity), *in vitro* α-glucosidase activity and cell cytotoxicity of pumpkin leaves. Steam blanching in plain water led to the highest retention of total phenolic content and reduced the loss of quercetin 3-glucoside 7-rhamnoside (Rutin), kaempferol 7-neohesperidoside, isoorientin 2″-O-rhamnoside, isorhamnetin-3-O-rutinoside, quercetin 3-galactoside, coumaroyl glucaric acid, isorhamnetin-3-galactoside-6″-rhamnoside, 2-caffeoylisocitric acid, quercetin 3-galactoside 7-rhamnoside by (3.04%), (7.37%), (10.65%), (10.97%), (14.88%), (16.1%), (16.73%), (18.88%), and (23.15%), respectively, and coumaroyl isocitrate increased by 14.92%. Candidate markers, 2-O-caffeoylglucaric acid, 2-(E)-O-feruloyl-D-galactaric acid, quercetin 3-galactoside 7-rhamnoside (rutin) and unidentified compounds ([(M-H) 677.28 and at RT 21.78] were responsible for the separation of the steam blanched samples in plain water from the other blanching treatments. Steam blanching in plain water increased the antioxidant capacity (FRAP and ABTS activity). There were no cytotoxic effect or inhibitory effect of α-glucosidase activity detected in the raw or blanched pumpkin leaves. Thus, this study recommends steam blanching in plain water for African cuisine, and confirms it is safe to consume pumpkin leaves frequently.

## Introduction

Pumpkins (*Cucurbita moschata* Duchesne ex Poir.) belong to the *Cucurbitaceae* family. Although pumpkins are indigenous to Mexico and Central America, due to their naturalization they are regarded as indigenous vegetables in the African region (1). Considered a healthy and functional vegetable, the consumption of pumpkin leaves is widespread in Korea, as well as in the Pacific Islands, India, and Bangladesh. Although pumpkins leaves play an important role in uplifting household food security among rural people in Sub-Saharan Africa (2), limited information is available on their functional properties. In addition, Cucurbita maxima Duchesne are commonly used for traditional medicinal treatments (3).

*Cucurbita moschata* leaves are large alternate, simple, ovate-cordate- sub-orbicular dark green leaves with a width of 20–25 cm and length of 25–30 cm (4). One cup portion (39 g) of pumpkin leaves contain 15 mg of calcium (Ca), 170 mg of potassium (K), 41 mg of phosphorus (P), 0.87 mg of iron (Fe), and 14 μg of folate. Compared to a cup portion of pumpkin leaves, lettuce leaves contain lower amount of Ca (14.04 mg), Fe (0.34 mg), K (75.66 mg), and P (11.31 mg) (5). In the Southern African region, pumpkin leaves are eaten as part of a maize-based diet as a relish, or the leaves and the tender shoots are included in soups or stews (6). In Korean cuisine, the pumpkin leaves are used to wrap a piece of meat filling (pork or nay meat) (“ssam”) (7).

Traditional vegetables are boiled mostly to improve their taste before consumption. Scientifically, blanching destroys the enzymes such as chlorophyllase and peroxidase, and prevents the loss of green color (8, 9). Traditionally, these vegetables are preserved in Africa during off seasons by adopting solar drying or shade drying; the vegetables are first blanched before being drying (10). Mkandawire and Masamba (10) showed that the vitamin C in the blanched vegetables improved when lemon juice in water was the blanching medium. Mkandawire and Masamba (10) used lemon juice as a blanching medium because the poor rural communities cannot afford commercial food additives. Raw pumpkin leaves showed stronger DPPH-, 2,2′-azino-bis (3-ethylbenzothiazoline-6-sulfonic acid)-radical scavenging activities, and ferric reducing antioxidant power (FRAP) compared to the fruit and the seeds (11). Polyphenols are antioxidants, known as antihypoglycemic agents, proven to lower the fasting blood sugar levels and the risk of type 2 diabetes (11). Dietary phenolic compounds inhibit the carbohydrate digestive enzymes, α-amylase and β-glucosidase (12) and reduce the re-absorption of glucose in the intestine. Type 2 diabetes is on the rise in Africa and is projected to increase to 41.5 million by 2035, and people between the ages of 40–59 are more vulnerable for type 2 diabetes (13).

No information is available on the changes in phenolic compounds present in pumpkin leaves during blanching or using lemon juice as a blanching medium the biological effects thereof. There is published research information available regarding different cooking methods on the antioxidant activity of some European vegetables (14), whilst the information on traditional vegetables is limited for consumers. Therefore, this study aims to provide the suitable blanching medium and the method that can minimize the loss of phenolic compounds for the commercial development of value-added functional plant-based diet products. The objective of this study was to investigate the effects of blanching methods such as (i) hot water dipping at 95°C in 5 or 10% lemon juice as blanching medium plain water as control, or (ii) steaming for 5 min using lemon juice solutions (5 or 10%) and plain water as the control on changes in phenolic metabolite and antioxidant properties in pumpkin leaves.

## Materials and Methods

### Chemicals

Methanol, acetic acid, formic acid, 3-(4, 5-dimethylthiazol-2-yl)-2, 5-diphenyltetrazolium bromide), hydrogen peroxide, disodium hydrogen phosphate heptahydrate; sodium dihydrogen phosphate monohydrate, enzyme porcine pancreatic solution, hydrochloric acid, ρ-nitrophenyl-α-D-glucoside solution, sodium carbonate, trolox, 2,2′-Azino-bis(3-Ethylbenzothiazoline-6-Sulfonic Acid), potassium persulfate, TPTZ [2,4,6-tris(2-pyridyl)-1,3,5-triazine], iron(III) chloride analytical standards (gallic, ferulic, vanillic, p-coumaric, elargic, gallic, 2.4 hydroxybenzoic, pyrogallol, protocatechuic, syringic acids, chlorogenic acid, catechin, epicatechin) at >95%, purity were purchased from Sigma Aldrich, Johannesburg, South Africa.

### Sample Preparation

Pumpkin leaves were selectively harvested at suitable maturity from the smallholders farming scheme, Tshiombo in Vhembe District, Limpopo Province. After harvest, leaves free from decay or damage or insect infestation were selected for the experiment. After washing in running tap water, transportation of the leaves to the laboratory was within 6 h, in cooler boxes at 10°C, after which they were stored at 5°C in the cold room for 24 h prior to processing. Following this, 50 g of leaves were subjected to different blanching treatments given below.

### Blanching Pre-treatment

There were 3 blanching treatments, (i) dipping in a 95°C water bath [thermostatically regulated water bath (PolyScience, Illinois, United States of America)], in plain water (control) or 5 or 10% lemon juice solutions (ii) steaming in a stainless-steel steamer pot for 5 min in plain water, or 5 or 10% lemon juice solutions (15).

After the blanching treatments, samples were cooled rapidly on ice to stop further biochemical reactions. Thereafter, the samples were freeze dried for untargeted and targeted phenolic metabolite analysis, *in vitro* antioxidant activity, α-glucosidase and cell cytotoxicity assay. Raw snap-frozen pumpkin leaves acted as a control, and each blanching treatment included 10 replicates for each analysis.

### Changes in Phenolic Metabolite Profile

Phenolic untargeted metabolite profile was identified and quantified using a Waters Ultra-Performance Liquid Chromatograph (UPLC), fitted with a Waters Acquity Photodiode Array Detector (PDA) and linked to a Synapt G2 quadrupole time of flight mass spectrometer (Waters, Milford, MA, USA), as described by Managa et al. (15) and Ndou et al. (16), without any modifications. Phenolic compounds were extracted from freeze dried pumpkin leaves (50 mg) that underwent different blanching treatments by ultrasonication in 70% aqueous ethanol. Phenolic compounds from pumpkin leaves that underwent different blanching treatments were extracted using ultrasonication of 50 mg freeze-dried samples in 70% aqueous ethanol. Concentrations of the phenolic compounds were determined using the reference calibrants catechin (LOD 1.414333, LOQ 4.286), epicatechin (LOD 5.105, LOQ 15.469), and rutin (LOD 3,294; LOQ 9.981), to quantify compounds based on the areas of their extracted mass chromatograms. The respective calibration curves are given in Supplementary Figure 1.

The LOD and LOQ values for TargetLynx software processed the obtained data, as described previously by Managa et al. (15) and Ndou et al. (16), and the concentration of phenolic compounds was expressed as mg kg^−1^.

### *In vitro* Total Antioxidant Capacities

The Ferric reducing antioxidant power (FRAP) assay was carried out according to the method of Managa et al. (15) for traditional leafy vegetables. Pumpkin leaf samples (0.2 g) were homogenized in 2 mL sodium acetate buffer (pH 3.6), and 15 μL of the leaf exact was used in this assay. FRAP assay was determined by mixing the leaf extract with 220 μl FRAP reagent solution [10 mmol L^−1^ TPTZ [2,4,6-tris(2-pyridyl)-1,3,5-triazine] acidified with concentrated HCl, and 20 mmol l^−1^ FeCl_3_]. Antioxidant power was expressed as mg of Trolox equivalent antioxidant content (TEAC) 100 g^−1^ FW.

2,2′-Azino-bis (3-Ethylbenzothiazoline-6-Sulfonic Acid) (ABTS) used for traditional vegetables by Managa et al. (15), was used for pumpkin leaves without any modifications. To prepare the ABTS^+^ stock solution, 7.4 mM ABTS^+^ solution and 2.6 mM potassium persulfate solution were mixed together (15) and held in darkness for 16 h at 25°C, then diluted with 0.1 mM phosphate buffer (pH 7.0) to obtain an absorbance at 734 nm (1.1 ± 0.002 units). The sample extract (15 μL), ABTS^+^ stock solution (285 μL), was mixed and held in darkness at 25°C for 2 h, then the absorbance was measured at 734 nm. Calibration curves were constructed using Trolox as the standard, and the antioxidant activity (ABTS assay) was expressed as μmg of TEAC g^−1^ FW.

### *In vitro* α-Glucosidase Inhibitory Activity

*In vitro* α-glucosidase inhibitory activity was determined according to the method described by Sagbo et al. (17) without any modifications using pumpkin leaf extract (5 μL). Pumpkin leaf extract was prepared at concentrations of 50–250 μg mL^−1^ mixed with 20 μL α-glucosidase solution (50 μg mL^−1^) in a 96-well-plate. Thereafter, 60 μL of potassium phosphate buffer (pH 6.8; 67 mM) was pipetted into the mixture in 96-well-plate and held at 35°C for 5 min, then 10 μL of 10 mM ρ-nitrophenyl-α-D-glucoside solution (PNPGLUC) was pipetted and held at 35°C for 20 min and finally, 25 μL of 100 mM Na_2_CO_3_ was pipetted and the absorbance was measured at 405 nm using a micro-plate reader (CLARIOstar Plus BMG Labtec, Lasec, Cape Town, South Africa). The absorbance was measured for both the leaf extracts, acarbose and the blank control (without α-glucosidase). The IC_50_ value (i.e., the concentration of pumpkin leaf extracts from different blanching treatment and media that resulted in 50% inhibition of maximal activity) was determined.

### Cell Cytotoxicity Using MTT Assay

Cell toxicity was measured using the method described by Moloto et al. (18) using (3-(4, 5-dimethylthiazol-2-yl)-2, 5-diphenyltetrazolium bromide) (MTT) cytotoxicity assay and C2C12 myoblast cell (skeletal muscle) line, without any modifications. Cells with cell density of 1 × 10^5^ cells mL^−1^ were cultured in a 96-well cell culture plate and thereafter treated with different concentrations (0.25–1.0 mg mL^−1^) of the pumpkin leaf extracts (extracted by ultrasonication of 50 mg freeze-dried samples using 70% aqueous ethanol) and incubated at 37°C for 24 h. Thereafter, an aliquot of 20 μL of 5 mg mL^−1^ MTT was pipetted into each well and incubated at 37°C for an additional 4 h to allow the conversion of MTT to the colored formazan. The untreated cells were included as control; H_2_O_2_ (0.25–2.5%) was used as positive control. Cell cytotoxicity was read at 570 nm using a microtitre-plate multimode detector (Promega-Glomax Multi-detection system, Madison, WI 53711, USA), using the formula below; the blank well-included only the medium.

$$\begin{array}{l} \%\text{ Viable cells }=\frac{abs\;sample-abs\;blank}{dabs\;control-abs\;blank}\;x100 \end{array}$$

### Statistical Analysis

A completely randomized design was adopted with 10 replicates per blanching treatment and the experiments repeated twice. A factorial analysis experiment was performed with different blanching treatments and the type of blanching media on phenolic compounds and *in vitro* antioxidant activity Two-way analysis of variance (ANOVA) was used to test the significant differences between the different blanching treatment and blanching media. Means were compared among the treatments by the least significant difference (LSD) test, with *p* < 0.05, using the Genstat statistical program for Windows, 13th Edition (2010) (VSN International Hempstead, UK). The UPLC-QTOF/MS data were used for unsupervised principal component analysis (PCA). Thereafter, supervised Orthogonal Projections to Latent Structures Discriminant Analysis (OPLS-DA) determined the compounds (candidate markers) responsible for the observed separation between the treatments and blanching media, as described previously by Managa et al. (15).

## Results and Discussion

### Changes in Non-targeted Phenolic Metabolites in Pumpkin Leaves During Blanching

Table 1 reveals the tentative identification of 24 phenolic compounds in pumpkin leaves using the UPLC-QTOF/MS. The majority (83.3%) of the phenolic compounds identified were glycosylated and the remaining portion were tetracarboxylic acids and derivatives (16.7%). Of the glycosylated phenolic compounds, flavonoid O-glycosides were the most abundant, constituting 41.7%, and simple phenolic glycosides made up 12.5% of the total phenolic compounds identified. The remaining glycosylated compounds identified were glucuronic acid derivatives, hydroxycinnamic acid glycosides and isoflavonoid O- glycosides, which constituted 12.5, 8.3, and 4.2% of the phenolic compounds identified, respectively.

**Table 1:** Tentative peak assignment of the phenolic metabolites present in pumpkin leaves subjected to different blanching treatments using different types of blanching medium.

**Compound**	**Retention time (min)**	**[M-H]^**−**^**	**M-H formula**	**Error (ppm)**	**MSE fragments**	**UV**	**Tentative identification**
1	8.03	315.0720	C_13_H_15_O_9_	0.32	108.0212 151.9972	314	Gentesic acid 5-O-glucoside
2	8.12	371.0605	C_15_H_15_O_11_	4.04	191.0259 209.0361	323	2-O-caffeoylglucaric acid
3	8.78	299.0762	C_13_H_15_O_8_	3.24	137.0240 93.0339	326	Pseudolaroside A
4	9.24	369.0478	C_15_H_13_O_11_	4.06	179.0393 188.9615 191.0090	326	2-O-Caffeoylhydroxycitric acid
5	9.56	385.0776	C_16_H_17_O_11_	0.00	85.0291 385.1417	320	2-(E)-O-feruloyl-D-galactaric acid isomer
6	10.08	285.0606	C_12_H_13_O_8_	3.5	108.02245	293	Diphenol glucuronide
7	10.23	341.0874	C_15_H_17_O_9_	1.17	135.0494 179.0366 221.0372 281.0751	331	1-O-Caffeoylglucose
8	10.69	355.0667	C_15_H_15_O_10_	1.12	163.0501 209.0332	314	Coumaroyl glucaric acid
9	11.15	385.0772	C_16_H_17_O_11_	1.04	85.02256 385.0714	320	2-(E)-O-feruloyl-D-galactaric acid isomer
10	12.03	325.0927	C_15_H_17_O_8_	0.62	146.0301 179.0382 119.0482	324	1-O-p-Coumaroyl-beta-D-glucose
11	12.95	593.1508	C_27_H_29_O_15_	0.67	269.0809	324	Luteolin 7-neohesperidoside
12	13.45	353.0502	C_15_H_13_O_10_	3.40	191.0261	331	2-caffeoylisocitric acid
13	15.52	447.0930	C_21_H_19_O_11_	0.67	147.0478 299.7440	270	Quercitrin
14	15.63	337.0552	C_15_H_13_O_9_	3.86	119.0431 191.0261	314	Coumaroyl isocitrate
15	16.67	769.2119	C_23_H_45_O_28_	−2.08	300.0241 315.0345	330	7-Methylquercetin-3-Galactoside-6″-Rhamnoside-3^‴^-Rhamnoside
16	16.74	367.0648	C_16_H_15_O_10_	6.27	173.0066 111.0049	329	Feruloyl isocitrate
17	16.80	609.1441	C_27_H_29_O_16_	3.28	147.0174 151.0100 162.9927 179.0179 300.0256 272.5696	255	Quercetin 3-galactoside 7-rhamnoside
18	17.00	609.1444	C_27_H_29_O_16_	2.79	151.0011 178.9964 273.0332 300.0256	255	Quercetin 3-glucoside 7-rhamnoside (Rutin)
19	17.27	431.0986	C_21_H_19_O_10_	−0.70	77.0398 93.0344	269	Genistin
20	17.96	593.1526	C_27_H_29_O_15_	−2.36	228.0345 256.0335 285.0390	264	kaempferol 7-neohesperidoside
21	18.59	593.1513	C_27_H_29_O_15_	−0.17	120.0294 447.0653 473.1074	265	Isoorientin 2″-O-rhamnoside
22	18.83	623.1610	C_28_H_31_O_16_	1.77	299.0212 315.0538	265	Isorhamnetin-3-Galactoside-6″-Rhamnoside
23	19.03	623.1600	C_28_H_31_O_16_	3.37	299.0239 315.0662	255	Isorhamnetin-3-O-rutinoside
24	19.93	503.1177	C_24_H_23_O_12_	3.58	177.0522 285.05307 315.5189	293	Pectolinarigenin 7-(6″-methylglucuronide)

Based on UPLC–QTOF/MS analysis, the ESI MS of peak 1 showed an [M-H]^−^ion at *m/z* 315.0657. In the MS/MS spectrum, a base peak ion was observed at *m/z* 108[M-H-162-45] due to subsequent loss of a sugar moiety and the cleavage of a carboxyl unit. Furthermore, a secondary peak was observed at *m/z* 152 [M-H-162]- due to loss of hexoside. Peak 1 was tentatively identified as gentisic acid 5-O-glucoside (18). In the first order mass spectrum of peak 2, an [M-H]^−^ ion was observed at *m/z* 371.0605. The MSE spectrum showed a base peak ion at *m/z* 209 [M-H-162]^−^ due to loss of a caffeoyl group. A secondary fragment, likely due to subsequent loss of a caffeoyl group, and a water molecule showing the presence of hydroxyl groups 191.0 [M-C_9_H_7_O_3_- H_2_O]^−^ was observed at *m/z* 191[M-H-162-18] (19, 20). The peak was tentatively identified as 2-O-caffeoylglucaric acid. The presence of glucaric acid was evident due to the presence of the product ion at *m/z* 209.0, as similarly reported by Abukutsa-Onyango (21) and Mathias and de Oliveira (22). The first order mass spectrum of peak 3 showed an [M-H]^−^ ion at *m/z* 299.0762. In the second order mass spectrum, a base peak ion was observed at *m/z* 137[M-H-162]^−^ due to loss of a glycosyl unit. A secondary peak was observed at *m/z* 93[M-H-162-44]^−^ due to subsequent cleavage of a glycosyl unit from the molecular ion and loss of the carbon dioxide from the aglycone. The loss of carbon dioxide was indicative of the presence of a carboxylic acid group in the aglycone (23). The tentative identification of peak 3 was as pseudolaroside A. In the ESI MS spectrum, peak 4 exhibited an [M-H]^−^ ion at 369.0404. The second order mass spectrum of this peak had fragments at *m/z* 179[M-H-191] due to loss of the hydroxycitric acid moiety, *m/z* 191[M-H-179] due to loss of the caffeic acid unit, 189 [M-H-207-18] due to subsequent loss of the caffeoyl moiety and a water molecule indicating the presence of hydroxyl groups. Therefore, Peak 4 was tentatively identified as 2-O-caffeoylhydroxycitric acid. Peak 6 exhibited an [M-H]^−^ ion at *m/z* 285.0606, in the MS/MS spectrum. A peak was observed at *m/z* 108[M-H-176] due to loss of a glucuronyl moiety (24), thus peak 6 was tentatively identified as diphenyl glucuronide.

Peak 7 exhibited a molecular ion at 341.0784 [M-H]^−^ in the MS/MS spectrum; peaks were observed at *m/z* 179[M-H-162] due to loss of a caffeoyl unit, m/z 135[M-H-179-28] due to subsequent loss of a glucose unit followed by cleavage of a carbon monoxide unit. Peak 7 was tentatively identified as 1-O-caffeoylglucose. In the ESI MS spectrum, peak 8 exhibited an [M-H]^−^ ion at *m/z* 355.0683. The second order mass spectrum showed peaks at *m/z* 209 and *m/z* 163. The peak at *m/z* 209[M-H-146] was due to loss of the coumaroyl moiety and the peak observed at *m/z* 163[M-H-192]^−^ was the result of the loss of the glucoyl moiety. This fragmentation pattern was used to tentatively identify the presence of coumaroyl glucaric acid. The ESI MS spectrum of peak 9 showed an [M-H]^−^ ion at *m/z* 385.0807. In the MS/MS spectrum a peak was observed at *m/z* 209[M-H-176]^−^ due to loss of the feruloyl group and in the MS^2^ at 146[M-H-176-18-15] due to loss of a water molecule showing the presence of a hydroxyl group and loss of a methyl group reflecting methylation of the aglycone. The peak was tentatively identified as 2-(E)-O-feruloyl-D-galactaric acid. In the first order mass spectrum, peak 10 showed a molecular ion at *m/z* 325.0927. In MSE spectrum of peaks observed at *m/z* 179, *m/z*119,04816 and *m/z*146. The peak at *m/z* 179.0382[M-H-146]^−^ was due to loss of a coumaroyl residue, the peak observed at *m/z* 146.0301[M-H-179]^−^ was due to loss of a glucose unit from the parent ion, and the peak observed at *m/z* 119.04816[M-H-179-28] due to subsequent loss of a coumaroyl residue followed by cleavage of a carbon monoxide unit. Peak 10 was tentatively identified as 1-O-p-coumaroyl-beta-D-glucose. The second order spectrum of peak 11 had peaks at *m/z* 269[M-H-146-179]^−^ due to subsequent loss of a rhamnosyl fragment and a glycose unit. The peak was tentatively identified as coumaroyl isocitrate. The MS/MS spectrum of peak 12 showed peaks at *m/z* 191[M-H-162]^−^ due to cleavage of the caffeoyl moiety; peak 12 was tentatively identified as 2-caffeoylisocitriic acid. In the ESI-MS/MS spectrum of peak 13 fragments were identified at *m/z* 147[M-H-301] from the cleavage of the aglycone from the parent ion. The peak at *m/z* 300[M-H-146] is the quercetin aglycone, which was due to loss of the rhamnose moiety. Consequently, peak 13 was tentatively identified as quercetrin, which is a glycoside of quercetin (quercetin-3-O-rhamnoside). The MS^2^ spectrum of peak 14 showed a peak at *m/z* 191[M-H-146]^−^ indicating the loss of the coumaroyl moiety, and another fragment was observed at *m/z* 119[M-H-191-28] from the loss of carbon monoxide from the coumaroyl moiety. Peak 14 was tentatively identified as coumaroyl isocitrate. The second order mass spectrum of peak 15 exhibited a peak at *m/z* 315[M-H-162-146-146] due to subsequent cleavage of the parent ion losing a hexose and two rhamnosyl fragments. A secondary peak characteristic of quercetin derivatives was observed at *m/z* 300, due to loss of a methyl group from the peak at *m/z* 315, thus revealing the presence of a methyl group attached to the quercetin moiety. Peak 15 was thus tentatively identified as 7-methylquercetin-3-galactoside-6″-rhamnoside-3^‴^-rhamnoside (xanthorhamnin). In the MS/MS spectrum, peak 16 had product ions at *m/z* 173[M-H-193] due to loss of a ferulic acid unit and *m/z* 111. This compound was tentatively identified as feruloyl isocitrate (25). Although the same fragmentation pattern was observed, another fragment at *m/z* 154 was not observed in this study. The MSE spectrum of peak 17 showed a base peak ion, which is the quercetin fragment at *m/z* 300.0256 [M-H-162-146] due to cleavage of the parent ion releasing the galactose and the rhamnose moieties. The fragment ion at *m/z* 300/301 is characteristic of all quercetin derivatives. The fragment observed at *m/z* 147[M-H-162-300] is the cleaved rhamnose moiety and the fragment observed at *m/z* 162[M-H-147-300] is the galactoside moiety. Other secondary fragments observed were mainly due to cleavage of the quercetin moiety and present in most quercetin derivatives; these were observed at *m/z* 273 due to loss of carbon monoxide from quercetin aglycone. Another secondary fragment observed at *m/z* 179 was a result of retrocyclization following fission on the C ring of quercetin (26). The fragment observed at *m/z* 151 was due to the loss of carbon monoxide from the *m/z* 179 fragment. Based on this fragmentation pattern, peak 17 was tentatively identified as quercetin 3-galactoside 7-rhamnoside. The MSE spectrum of peak 18 showed a base peak ion at *m/z* 300[M-H-146-162]^−^ due to subsequent loss of the rhamnose and glycosyl units from the parent ion, revealing that the compound was di-glycosylated. Secondary fragments were observed mainly due to fragmentation of the quercetin moiety at *m/z* 273[M-H-308-28] due to subsequent loss of the diglycosyl unit and release of a carbon monoxide from the quercetin aglycone. Another at *m/z* 179 was a result of retro cyclization following fission on the C ring of quercetin. Fernández-Poyatos (19) observed a similar fragmentation pattern for rutin. Peak 18 was tentatively identified as quercetin 3-glucoside 7-rhamnoside (rutin). In the MS/MS spectrum, peak 19 exhibited a peak at *m/z* 93[M-H-179] due to cleavage of a chromone moiety. Another fragment observed at *m/z* 77 [M-H-179-18] was attributed to subsequent loss of the chromone moiety and cleavage of a water molecule, revealing the presence of a hydroxyl group on a benzene ring. As a result, peak 19 was tentatively identified as genistin (7 hydroxyisoflavone). The ESI-MS/MS spectrum of peak 20 exhibited peaks at *m/z* 285[M-H-146-162]^−^ due to subsequent loss of a rhamnosyl and a glucosyl unit from the parent ion. The fragments observed at *m/z* 256 and *m/z* 228 were due to fragmentation of the kaempferol moiety (27). The ESI MS spectrum of peak 21 had an [M-H]^−^ ion observed at *m/z* 593.1512. In the second order spectrum, the base peak ion observed at *m/z* 473.1074[M-H-120] and the peak observed at *m/z* 120[M-H-473]^−^ were attributed to intra-glycoside fragmentation of glucose linked to the carbon atom of flavone in the isoorientin moiety. Secondary fragments were observed at *m/z* 447[M-H-146] due to the loss of the rhamnose moiety (22). Peak 21 was thus tentatively identified as Isoorientin 2″-O-rhamnoside. In the MS/MS spectrum of peak 22, a base peak ion was observed at *m/z* 315.0538[M-H-308]^−^; Lei et al. (28) demostrated a similar fragmentation pattern. The loss of 308 indicated the compound was di-glycosylated having a hexose and a deoxyhexose with the ion at 315 being the aglycone ion. Furthermore, the secondary peak observed at *m/z* 299[M-H-308-15]^−^ was attributed to the loss of a methyl group, revealing the aglycone moiety was methylated. Peak 22 was tentatively identified as isorhamnetin-3-galactoside-6″-rhamnoside. Peak 23 exhibited a similar fragmentation pattern to that of peak 22, revealing the compounds belonged to the same class. The second order spectrum of peak 23 showed a base peak ion at *m/z* 315[M-H-146-162]^−^ due to subsequent loss of a deoxyhexose and a hexose sugar, with the base peak ion being the aglycone. Secondary fragments were also observed at m/z 299[M-H-308-15] due to cleavage of a methyl group from the aglycone moiety showing the presence of a methyl group in the aglycone. Peak 23 was tentatively identified as isorhamnetin-3-O-rutinoside and similar pattern of fragmentation was identified in the study as Lei et al. (28). The MSE spectrum of peak 24 had a peak at *m/z* 315[M-H-191] from cleavage of the parent ion to produce the glycone and the aglycone moiety. A peak observed at *m/z* 177 was due to demethylation of the glycone moiety. Another peak ion observed at *m/z* 285 was identified as a fragment of methylated naringenin. Thus, peak 24 was tentatively identified as pectolinarigenin 7-(6″-methylglucuronide). The MS spectra of the compounds listed in Table are given in Supplementary Table 1.

Table 2 illustrates the changes in different untargeted phenolic compounds and total phenolic compounds during different blanching treatments using plain water or lemon juice as the blanching medium. Overall, the total phenolic compounds were highest (1,457.1 mg kg^−1^) in raw pumpkin leaves compared to cooked samples. This illustrates that during the process of cooking, polyphenols were broken down from the leaf tissue; a similar observation was reported by Gunathilake et al. (29). Water bath blanched leaves in plain water or 5 or 10% lemon juice, showed 521.4, 530.6, 658.7 mg kg^−1^ total phenolic compounds, respectively, indicating the loss of total phenolic compounds. Steam blanching in 5 or 10% lemon juice showed 885.4 mg kg^−1^, 906.4 mg kg^−1^ of total phenols, respectively, which was higher than the levels observed during water bath blanching. Compared to all blanching treatments, steam blanching in plain water helped to retain the total phenolic content to 1,299.2 mg kg^−1^. Steaming in plain water showed the lowest 10.83% loss of total phenolic compounds compared to other cooking methods with reference to the raw leaves. In contrast, the following steamed traditional vegetables, *Sesbania grandiflora* (“kathurumurunga”), *Passiflora edulis* (“passion fruit”), and *Olax Zeylanica* (“mella”) leaves, showed significant loss of polyphenols compared to their raw leaves (29). Turkmen et al. (30) reported that blanching in hot water reduced the polyphenolic content by only 12–26% in spinach, swamp cabbage, kale, shallots, and cabbage; however, Kao et al. (31) showed a significant increase in polyphenols in Thai basil and sweet potato leaves, during boiling for 1–5 min.

**Table 2:** Influence of different blanching treatments and media on different phenolic components of pumpkin leaves.

	**Raw pumpkin leaves**	**Water bath blanching–Plain water**	**Loss %**	**Steam blanching–Plain water**	**Percentage loss %**	**Water bath blanching −5% lemon juice**	**Percentage loss %**	**Water bath blanching −10% lemon juice**	**Percentage loss %**	**Steam blanching −5% lemon juice**	**Percentage loss %**	**Steam blanching 10% lemon juice**	**Percentage loss %**
**mg kg**^**−1**^													
2-O-Caffeoylglucaric acid	89.5 ± 6.4^*^^a^	9.7 ± 1.2^d^	89.16	30.6 ± 2.6^bc^	20.72	20.6 ± 3.8^c^	76.98	34.4 ± 1.4^bc^	61.56	37.5 ± 2.6^bc^	58.1	44.8 ± 2.1^b^	49.94
Coumaroyl glucaric acid	55.3 ± 3.2^a^	3.1 ± 0.6^c^	94.39	45.4 ± 2.0^a^	17.90	5.5 ± 0.1c	90.05	9.3 ± 0.6^c^	83.18	31.0 ± 3.1^b^	43.94	27.6 ± 1.6^b^	50.09
2-(E)-O-feruloyl-D-galactaric acid	52.1 ± 3.2^a^	9.7 ± 0.3^b^	81.3	43.7 ± 2.6^a^	16.1	9.3 ± 0.1b	82.14	13.9 ± 1.5^b^	73.32	42.0 ± 2.9^a^	19.38	46.0 ± 3.4^a^	11
2-caffeoylisocitric acid	38.6 ± 1.9^b^	3.9 ± 1.2^d^	89.89	72.6 ± 1.0^a^	18.88	11.6 ± 1.0^cd^	69.94	15.9 ± 0.8^c^	58.80	21.4 ± 1.0^c^	44.55	25.8 ± 3.5^bc^	33.16
Coumaroyl isocitrate	47.9 ± 2.8^b^	6.9 ± 1.2^c^	85.59	56.3 ± 2.5^a^	−14.92	8.2 ± 0.6c	82.88	9.6 ± 1.6^c^	79.95	43.8 ± 3.3^b^	8.55	42.9 ± 2.6^b^	10.43
Quercetin 3-galactoside 7-rhamnoside	149.9 ± 3.7^b^	59.3 ± 3.1^d^	60.44	115.2 ± 1.1^b^	23.15	52.1 ± 1.6^d^	65.24	242.9 ± 3.1^a^	30.97	86.5 ± 6.0^c^	42.29	92.6 ± 1.8^c^	38.22
Quercetin 3-glucoside 7-rhamnoside (Rutin)	351.9 ± 2.9^a^	222.3 ± 2.2^c^	36.82	341.2 ± 2.7^a^	3.04	189.4 ± 1.6^c^	46.17	57.2 ± 2.5^d^	26.34	267.9 ± 1.2^b^	23.87	271.1 ± 2.1^b^	22.96
Quercetin 3-galactoside	112.2 ± 2.7^a^	42.4 ± 1.7^d^	62.21	95.5 ± 1.1^b^	14.88	41.9 ± 1.2^d^	62.66	42.4 ± 3.0^d^	62.65	58.5 ± 2.3^c^	47.86	54.9 ± 1.3^cd^	51.06
Kaempferol 7-neohesperidoside	123.4 ± 1.4^a^	34.8 ± 3.7^d^	71.79	114.3 ± 2.7^a^	7.37	37.7 ± 2.3^cd^	69.36	55.2 ± 2.6^c^	55.26	60.9 ± 2.7^bc^	50.64	75.2 ± 4.6^b^	39.05
Isoorientin 2″-O-rhamnoside	182.1 ± 2.7^a^	41.2 ± 2.5^e^	77.37	162.7 ± 3.2^b^	10.65	42.0 ± 15.4^e^	76.93	63.8 ± 2.9^d^	64.96	89.0 ± 3.3^c^	51.12	90.6 ± 1.4^c^	50.24
Isorhamnetin-3-galactoside-6″-rhamnoside	80.1 ± 2.1^a^	18.1 ± 3.7^d^	77.4	66.7 ± 9.7^b^	1.7	28.6 ± 0.8^cd^	3.2	29.9 ± 1.9^c^	62.67	36.8 ± 2.0^c^	54.05	34.9 ± 1.7^c^	56.42
Isorhamnetin-3-O-rutinoside	174.1 ± 1.7^a^	70.0 ± 2.3^d^	59.79	155.0 ± 2.4^b^	10.97	83.7 ± 2.2^d^	51.92	84.2 ± 1.2^d^	51.63	110.1 ± 1.7^c^	36.76	100.0 ± 2.6^c^	42.56
Total phenolic compounds	1,457.1 ± 4.1^a^	521.4 ± 3.4^d^		1,299.2 ± 2.5^b^		530.6 ± 3.1^d^		658.7 ± 2.8^d^		885.4 ± 2.9^c^		906.44 ± 1.7^c^	

*Rows with similar alphabetic letter are not significantly different at p < 0.05 according to Fisher's LSD test*.

* *Standard deviation (n = 3, cumulated sample of 10 makes 1 n replicate)*.

It is also evident from our study that the type of blanching treatment used, compared to the type of blanching medium, profoundly influenced the retention of total phenolic compounds in pumpkin leaves. Compared to all 12 phenolic compounds listed in Table 2, quercetin 3-glucoside 7-rhamnoside (Rutin) showed the least amount of loss during water bath (36.82%) and steam blanching (3.04%) in plain water and water bath blanching in lemon juice (5%) (46.17%) or (10%) (26.34%). The observed loss of rutin during blanching can be attributed due to its reduced sensitivity to thermal treatment due to the presence of glucose in the molecular structure (32). The loss of 2-(E)-O-feruloyl-D-galactaric acid during steam blanching with different blanching media (lime juice or plain water) varied insignificantly, between 11.7 and 19.38%. Galactaric esters of ferulic acid [2-(E)-O-feruloyl-D-galactaric acid] are highly soluble due its polar nature, and its loss is reduced to 16.1% in steam blanched leaves in plain water. In addition, the acidic pH could have retained the stability of the molecule and prevented further degradation of this compound to free phenolic acid (ferulic acid) (33) during steam blanching in 5% and 10% lime juice.

The concentration of coumaroyl glucaric acid ester and coumaroyl isocitrate was 55.3 and 47.9 mg kg ^−1^ in raw pumpkin leaves, respectively. It is interesting to note that coumaroyl glucaric acid was reduced by 17.90%, whilst coumaroyl isocitrate gained by −14.92% after steam blanching in plain water. This was probably because coumaroyl glucaric acid ester underwent hydrolysis and the hydroxyl-cinnamic acid (*p*-coumaric acids) could have formed conjugates via esterification between isocitric acid (34). Similarly, concentrations of 2-O-caffeoylglucaric acid and 2-caffeoylisocitric acid in raw pumpkin leaf was 89.5 and 38.6 mg kg^−1^, respectively. After steam blanching in plain water, the concentration of caffeoylisocitric acid increased to 72.6 mg kg^−1^ and 2-O-caffeoylglucaric acid was reduced to 30.6 mg kg^−1^ (Table 2). Kaempferol 7-neohesperidoside reduced loss by 7.37% during steam blanching in plain water, and the concentrations of this compound reduced non-significantly compared to the raw leaves, possibly because acylation may have provided increased resistance to heat treatment (35). Conversely, 10.65% of isoorientin 2”-O-rhamnoside, the c-glycosyl flavonoids was lost during steam blanching in plain water, whilst steam blanching in 5 or 10% lemon juice resulted in ~50% loss. Likewise, steaming pumpkin leaves in plain water led to 10.97% loss of isorhamnetin-3-O-rutinoside, whereas steaming in 5 or 10% lemon water (acidic pH) resulted in greater loss by 36.76 and 42.56% respectively. Phenolic compounds have different thermal stability according to their molecular structure (32, 35, 36) and their degradation depends on the temperature, pH and duration (time) (27, 35, 37, 38). For the stability of flavonoids in a food matrix, the hydroxyl group in position 3 of the C ring, with glycosylation and sugar moiety, was reported to block its degradation during thermal treatment (32). Steamed celery or parsley leaves in plain water showed significantly greater amounts of flavonoid glycosides (38). Therefore, the blanching treatments were optimized in this study to minimize the flavonoid derivatives in pumpkin leaves that may enhance the bioavailability and efficacy.

Moist cooking of nightshade in plain water in water bath at 95°C caused loss of different phenolic compounds (39). However, Managa et al. (39) reported lower concentrations of total phenolic compounds in raw leaves compared to the steamed blanched leaves in water or 5% lemon juice. Conversely, an opposite trend was noted in pumpkin leaves, where the raw leaves showed the highest phenolic components and total phenolic content. In contrast to our observation, in African nightshade leaves total phenolic compounds and different phenolic components increased during boiling in water (39), probably due to higher extractability of the phenolic compounds from the cellular matrix of the leaves (40). During blanching in water bath with plain water or 5 or 10% lemon juice, the different phenolic components listed in Table 2 had probably been released into the boiling water due to the heat mediated rupture of the leaf cell wall (41). Phenolics are polar compounds highly soluble in water (42) and during steaming they are retained on the leaf surface. The structural property of the cell wall of the different vegetables determines the ability of the cell wall to withstand the thermal blanching treatment and to retain phenolic compounds within the cells (43). Due to the higher temperatures, blanching was reported to degrade the polyphenol oxidase enzymes that use the phenols as substrates during browning reaction, and steam treatments in broccoli, compared to boiling, effectively reduced the peroxidase activity (44). This could be the reason for the higher retention of total phenol compounds and different phenolic components in steamed blanched pumpkin leaves in this study, compared to the other blanching treatments.

### Multivariate Analysis

Principal Component Analysis (PCA) analyses was conducted as it is difficult to interpret large quantities of generated data, so this technique helped to reduce the dimensionality of the original data set, improving the interpretation, and reducing the loss of valuable information, and therefore PCA occurred in this study, as reported by Biancolillo and Marin**i** (45). PCA helps in data compression, while reducing the loss of information (45), in an unsupervised nature. In addition, the PCA loading scores helped to present the data, exhibiting the possible trends in the presence of clusters (45), and reveals only the group structure.

**Figure 1:**
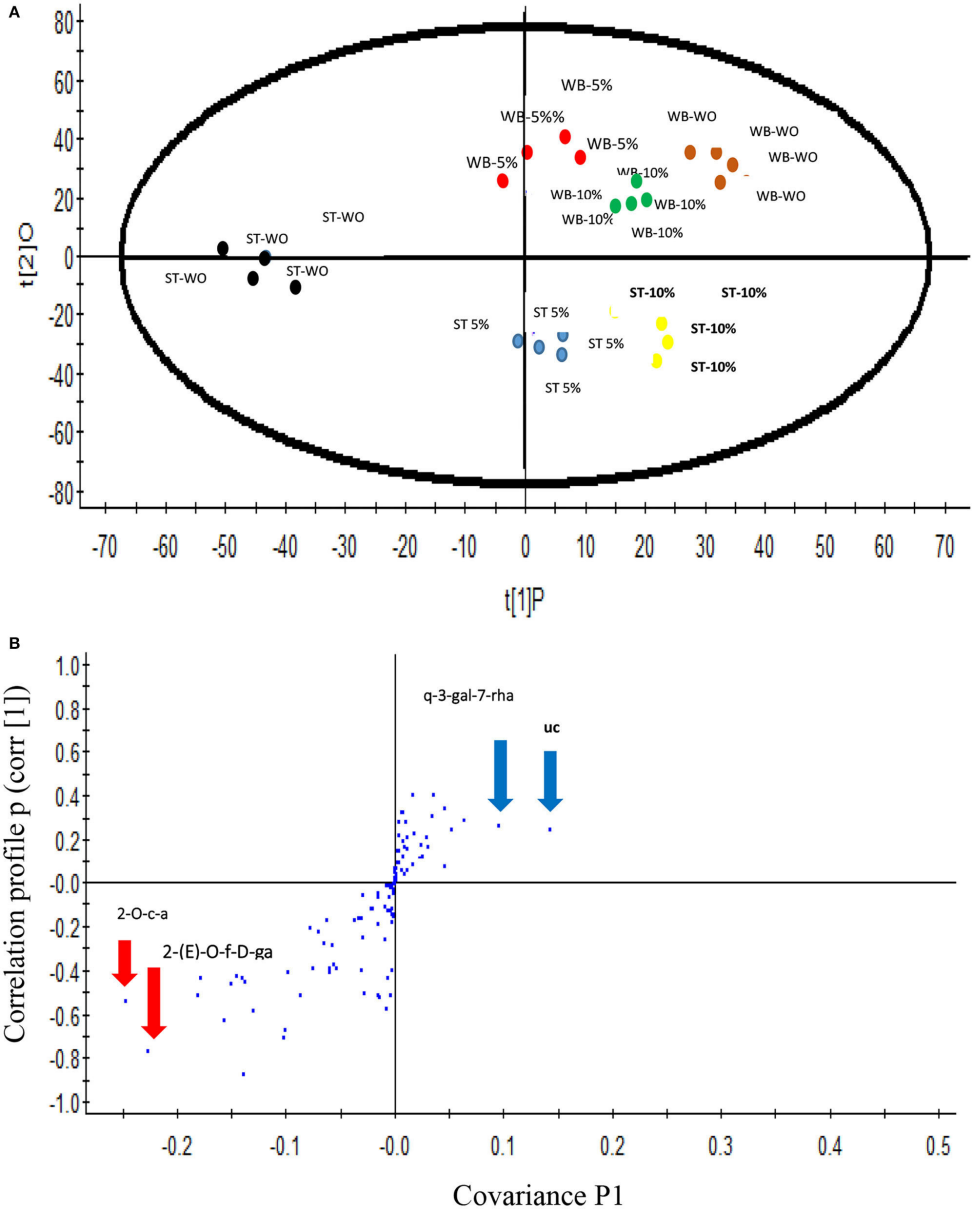
**(A)** Score plot of Principal Component analysis (unsupervised) based on UPLC–Q-TOF/MS spectra of the blanched pumpkin leaves. ST WO-Steam blanching in plain water at 95°C for 5 min; WB-WO water bath blanching in plain water; WB- 5%–Water bath blanching in 5% Lemon water at 95°C for 5 min; WB- 10% -Water bath blanching in 5% Lemon water at 95°C for 5 min. **(B)** Score plot of orthogonal partial least squares discriminant analysis of UPLC–Q-TOF/MS spectra of steam blanched pumpkin leaves in plain water and other samples underwent water bath blanching in plain water or 5 or 10% lemon water. Each sample set includes 4 replicates. 2-O-caffeoylglucaric acid (2-O-c-a), 2-(E)-O-feruloyl-D-galactaric acid [2-(E)-O-f-D-ga], quercetin 3-galactoside 7-rhamnoside (q-3-gal-7-rha), unidentified compound (uc) [(M-H) 677.28 and at RT 21.78].

The differences in distribution of the non-targeted phenolic metabolic profiles of the pumpkin leaves that underwent different blanching treatments was demonstrated using an unsupervised (PCA) approach, using the data set obtained by the UPLC–Q-TOF/MS analysis. Figure 1A illustrates the PC 1 and PC 2, explaining the 38 and 16.4% of the variance and showing good statistical separation among the different blanching treatments. The PCA plot showed 3 district clusters based on the phenolic metabolites shown by different blanching treatments and media used in this study. Samples that underwent steam blanching in plain water were separated from other two which were blanched in a water bath or steam using 5 or 10% lemon juice. Also, water bath blanching and steam blanching were further separated in PCA. An Orthogonal Projections to Latent Structures Discriminant Analysis (OPLS-DA) was performed for metabolomics data to analyze the multivariate data to establish a quantitative relationship between the different blanching treatments and the phenolic compounds (46), to identify the biomarker candidates responsible for separation between the blanching treatments via a more mathematically supervised manner using the description of the algorithms. The OPLS-DA provides more information than the PCA and allows better expose separations between classes in clusters. Therefore, well-separated PCA can guide PLS classification with greater possibility of providing the information for the compounds responsible for the observed separation of the clusters (46) in this study.

**Figure 2:**
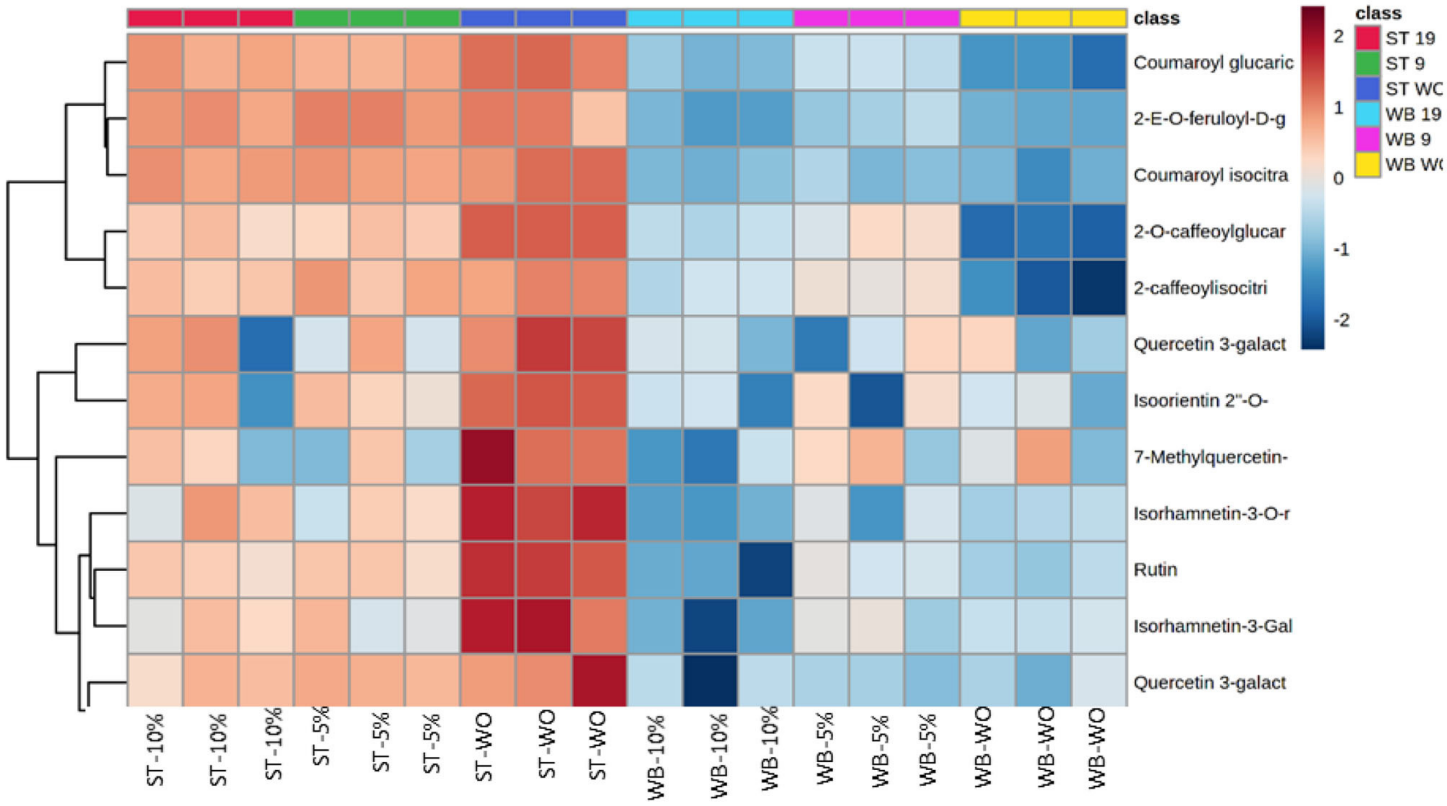
Heat map of twelve phenolic metabolites quantified in blanched pumpkin leaves using plain water or 5 or 10% lemon water organized in hierarchical clustering.

The values of *R*^2^ X and *Q*^2^ of cross-validation in OPLS-DA score plot for pumpkin leaves were 0.972 and 0.957, respectively, and provided reliable fitness. Figure 1B shows the S-plot; the upper right quadrant showed the phenolic compounds that were present in pumpkin leaves at higher concentrations in steam and water blanching treatments in 5 or 10% lemon water, whilst the lower left quadrant demonstrated the compounds that were higher in concentration during steam blanching in plain water. Phenolic compounds, 2-O-caffeoylglucaric acid, 2-(E)-O-feruloyl-D-galactaric acid, quercetin 3-galactoside 7-rhamnoside (rutin) and unidentified compounds [((M-H) 677.28 and at RT 21.78)] were identified as the candidate markers responsible for the separation of the steam blanched samples in plain water from the other blanching treatments. Exact Mass/Retention Time pairs responsible for the separation of steam blanched pumpkin leaves in plain water from the other blanching treatments is given in Table 3. The heat map shown in Figure 2 illustrates the intensity of different non-targeted phenolic metabolites in pumpkin leaves to the different blanching treatments. The heat map includes the clustergrams, the row data represented the concentration of phenolic metabolites relating the columns of variables the blanching treatments and the type of media used. Color block was used to express the intensity of the influence of the treatment; red box representing higher concentrations and blue box indicating the metabolites at a lower concentration. The heat map illustration clearly showed that generally, the steamed leaves contained the highest concentration of the 12 phenolic metabolites (2-O-caffeoylglucaric acid, coumaroyl glucaric acid, 2-(E)-O-feruloyl-D-galactaric acid, 2-caffeoylisocitric acid, coumaroyl isocitrate, quercetin 3-galactoside 7-rhamnoside, quercetin 3-glucoside 7-rhamnoside (rutin), quercetin 3-galactoside, isoorientin 2″-O-rhamnoside, isorhamnetin-3-galactoside-6″-rhamnoside, isorhamnetin-3-O-rutinoside) compared to the water bath blanching. Among the steam treatments, pumpkin leaves steamed in plain water showed the higher concentrations of the 12 phenolic metabolites.

**Table 3:** Exact Mass/Retention Time pairs responsible for the separation of steam blanched pumpkin leaves in plain water from the other blanching treatments.

**Primary ID**	**Retention time**	**Mass**	**p[1]P**	**p(corr)[1]P**	**Factor of Change**	**Steam blanching**	^*^Other blanching treatment
2-O-Caffeoylglucaric acid	8.12	371.0605	−0.227376	−0.769076	3.8	107.24	28.2241
2-(E)-O-Feruloyl-D-galactaric acid	9.56	385.0772	−0.138788	−0.872095	−21,2574,480.0	38.9443	−1.83203e-007
Quercetin 3-galactoside 7-rhamnoside (rutin)	17.00	609.1444	0.434539	0.671667	1.7	422.558	731.997
Unidentified compound	9.74	677.28	−0.156446	−0.631567	3.4	91.3982	26.6321

* *Other blanching treatments—Steam blanching in 5 or 10% lemon juice; water bath blanching in plain water*.

### *In vitro* Antioxidant Capacity and α-Glucosidase Activities and Cytotoxic Effect

Table 4 presents the results from *in vitro* antioxidant capacity, α-glucosidase activity, and cytotoxic effect. Antioxidant capacity (FRAP and ABTS assay) was highest in raw pumpkin leaves, whilst amongst the 6 blanching treatments adopted in this study, steaming in plain water showed significantly highest antioxidant capacity (FRAP and ABTS assay). The strongest antioxidant capacity (FRAP and ABTS assay) of pumpkin leaves that underwent blanching, based on FRAP (1.25 μmol TEAC g^−1^ FW) and ABTS (1.51 μmol TEAC g^−1^ FW) assay, were found in steamed blanched leaves in plain water followed by steam blanched leaves using 10% lemon water as the blanching medium. However, water bath blanched leaves using 5 or 10% lemon juice as the blanching medium showed the least antioxidant capacity. This indicated that steaming at 95°C helped to retain the antioxidant capacity of the pumpkin leaves. The observed trend in antioxidant capacity corresponds to the cumulative yield of the phenolic compounds shown in Table 2, and the released free phenolic compounds could have had a positive effect on the antioxidant activity in pumpkin leaves steam blanched in plain water, probably due to the destruction of the cell wall and cellular components releasing the antioxidants (30). As in our observation, steaming in water reduced the total antioxidant capacity of traditional vegetable *O. zeylanica* and Nightshade (*Solanum retroflexum* Dun) leaves compared to its raw form (29, 39). Steaming in water also preserved the antioxidant properties of green bean varieties (47). Conversely, Gunathilake et al. (29) also revealed that steamed leaves of traditional vegetable *P. edul* showed 10% higher antioxidant capacity compared to the fresh leaves. Therefore, there are discrepancies related to the preservation of antioxidant activity during different blanching and other cooking methods; blanching of Nightshade in plain water showed similar reduction in antioxidant activity as pumpkin leaves in this study (39). The highest correlation between total phenolic compounds and FRAP or ABTS activity was established by Qader et al. (48) and Augusto et al. (49).

**Table 4:** Effects of different house domestic cooking methods on *in vitro* antioxidant capacity and cytotoxic effect of blanched pumpkin leaves.

**Treatments**	**FRAP (μmol TEAC g^**−1**^ FW)**	**ABTS (μmol TEAC g^**−1**^ FW**	**Inhibition of α-glucosidase activity IC_**50**_ (μg mL^**−1**^)**	**Cell viability IC_**50**_ (μg mL^**−1**^)**
Raw	1.41 ± 0.29a	1.52 ± 0.12^a^	21.31 ± 0.10^e^	46.22 ± 0.81^b^
Water bath: plain water	0.37 ± 0.27^e^	0.47 ± 0.20^d^	26.54 ± 0.47^d^	13.65 ± 0.50^d^
Water bath: 5% lemon juice	0.91 ± 0.52^c^	0.82 ± 0.21^c^	28.12 ± 0.84^c^	15.29 ± 0.34^d^
Water bath: 10% lemon water	0.67 ± 0.41^d^	1.38 ± 0.81^b^	27.69 ± 0.97^cd^	12.56 ± 0.34^d^
Steaming: plain water	1.25 ± 0.20^b^	1.51 ± 0.54^a^	30.89 ± 0.11^a^	25.62 ± 0.76^c^
Steaming: 5% lemon juice	0.96 ± 0.73^c^	1.34 ± 0.23^b^	31.24 ± 0.76^a^	25.33 ± 0.83^c^
Steaming: 10% lemon juice	1.02 ± 0.30^c^	1.41 ± 0.43^b^	29.51 ± 0.50^b^	13.08 ± 0.21^d^
Hydrogen peroxide				70.17 ± 0.15^a^
Acarbose			18.22 ± 0.11^*f*^	

*Means followed by the same letter within the column are not significantly different at p < 0.05 according to Fisher's LSD test*.

Cytotoxic effects of leaf extracts of raw and blanched pumpkin leaves on C2C12 myoblast cell line are presented in percentage cell viability. All blanching treatments, irrespective of the type of blanching media used and the raw pumpkin leaves, showed the absence of inhibitions on cell viability at 50%, indicating the absence of toxicity, and the control (H_2_O_2_) showed the highest toxicity. Cytotoxic evaluation is important to screen for toxic effects, and during blanching, some of these compounds could have probably denatured or been removed (50). Raw and blanched pumpkin leaves illustrated the lowest inhibition activity with IC_50_ ranging from 21.31 to 30.89 μg mL^−1^, indicating that the pumpkin leaves are weak inhibitors of α-glucosidase. However, the inhibitory effect of raw pumpkin leaves on α-glucosidase activity is not comparable with the commercial inhibitor acarbose, and the fruit pulp and seeds of pumpkin (*C. maxima*) reportedly had the active hypoglycemic components (50, 51).

## Conclusion

The study indicated that the blanching methods and the type of blanching media affected the dietary phenolic compounds, and antioxidant capacity of pumpkin leaves. More specifically, steam blanching in plain water improved the retention of phenolic compounds and antioxidant capacity than all other adopted blanching methods. Use of lemon water during steam blanching is not recommended. All blanching methods and raw leaves showed the absence of cytotoxicity, and were safe for frequent consumption. This information is useful for food manufacturers and chefs, however further studies are needed to see if the leaves of other pumpkin cultivars and from different geographical regions offer health benefits.

## Data Availability Statement

The raw data supporting the conclusions of this article will be made available by the authors, after the approval of the University Scientific committee.

## Author Contributions

FM performed the experiment, generated the data, and wrote some parts of this manuscript. TS visualized and validated the data for phenolic compounds, interpreted the chromatogram and wrote that part of the article. JS was responsible for the antidiabetic activity and data. RS provided the editorial support. DS conceptualized the research, supervised the FM and improved the article further. All authors contributed to the article and approved the submitted version.

## Conflict of Interest

The authors declare that the research was conducted in the absence of any commercial or financial relationships that could be construed as a potential conflict of interest.
