# Supplementary material for: Changes in Phenolic Metabolites and Biological Activities of Pumpkin Leaves (Cucurbita moschata Duchesne ex Poir.) During Blanching
Source: Front Nutr. 2021 Mar 15;8:641939. doi: 10.3389/fnut.2021.641939 (PMC8005549; doi:10.3389/fnut.2021.641939)
Supplement: Supplementary file 1 [file Data_Sheet_1.PDF]

Supplementary Table 1 Ms spectra of the compounds

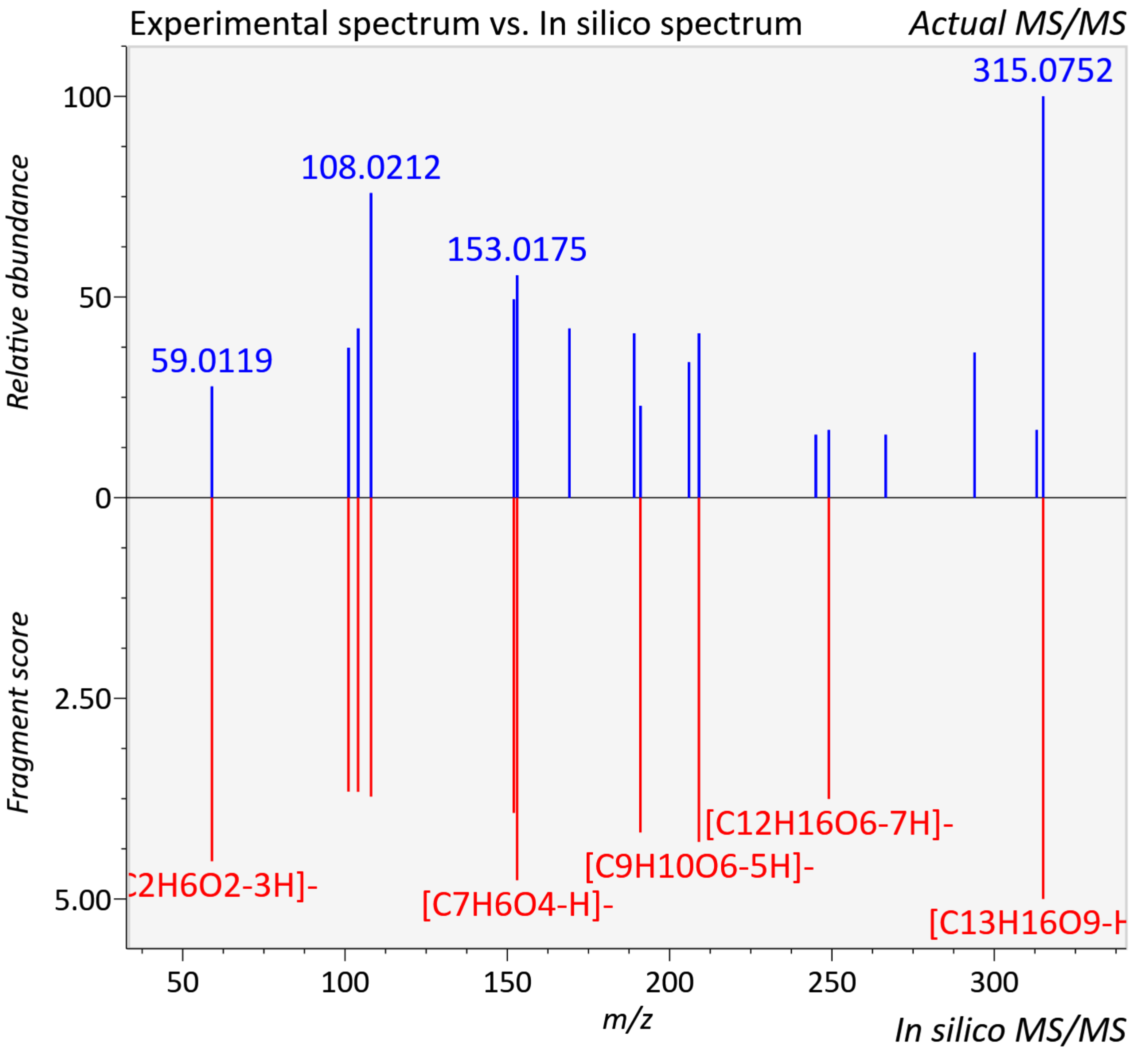

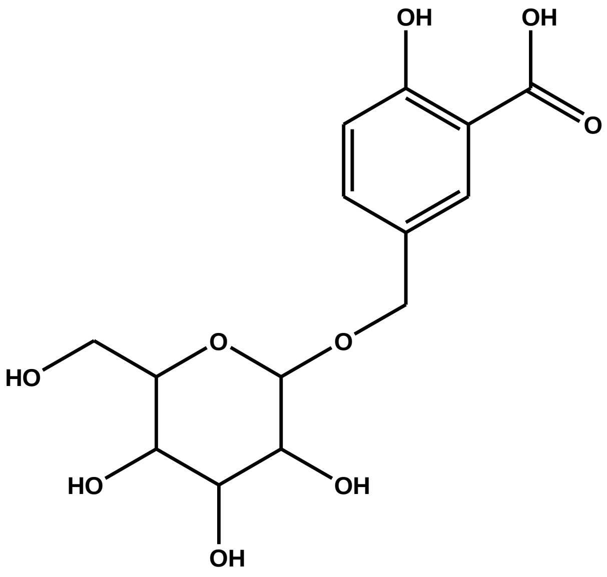

Supplementary Figure……MS/MS spectra for gentesic acid 5-O-glucoside

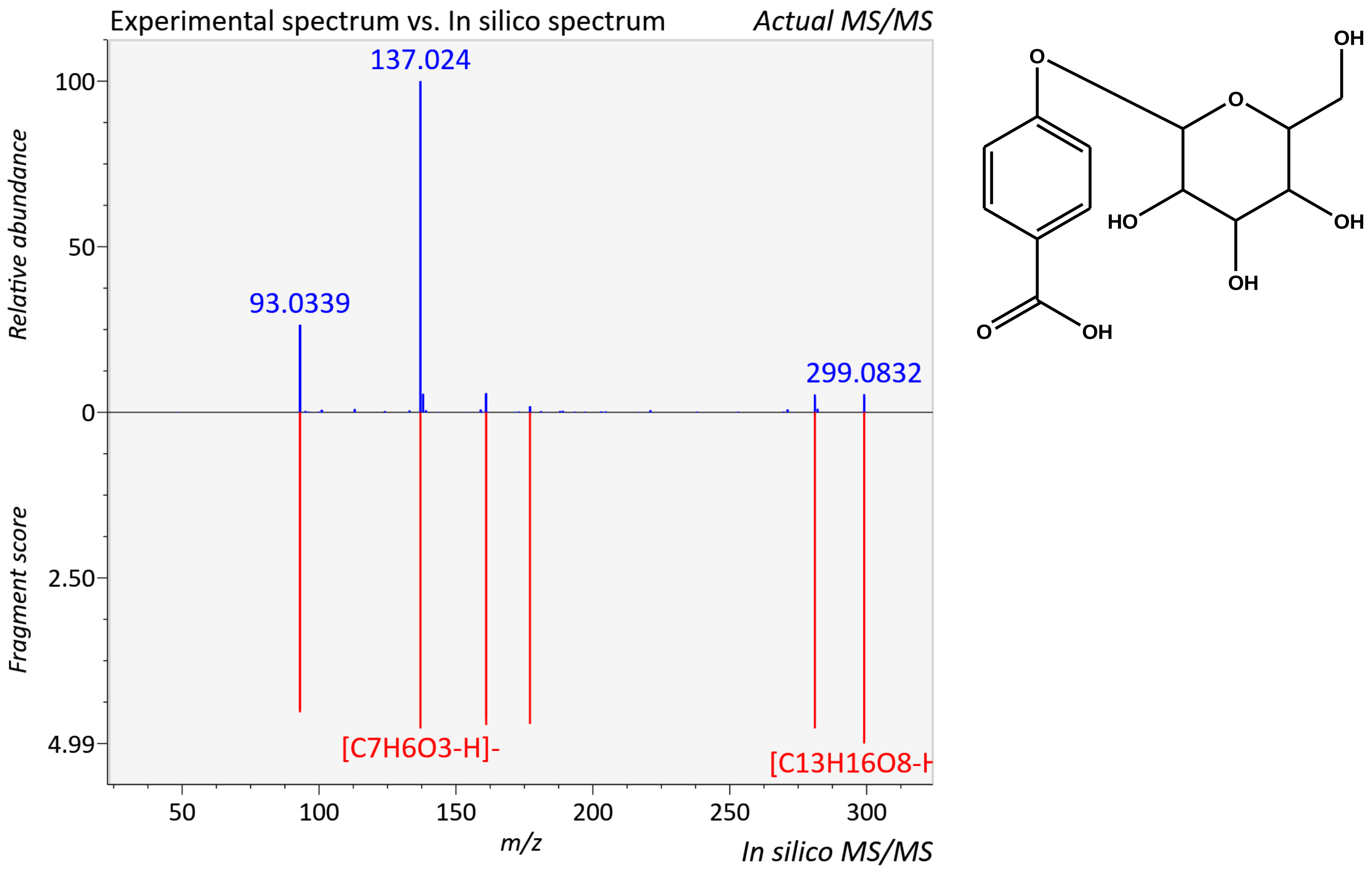

Supplementary Figure…MS/MS spectra for Pseudolaroside A

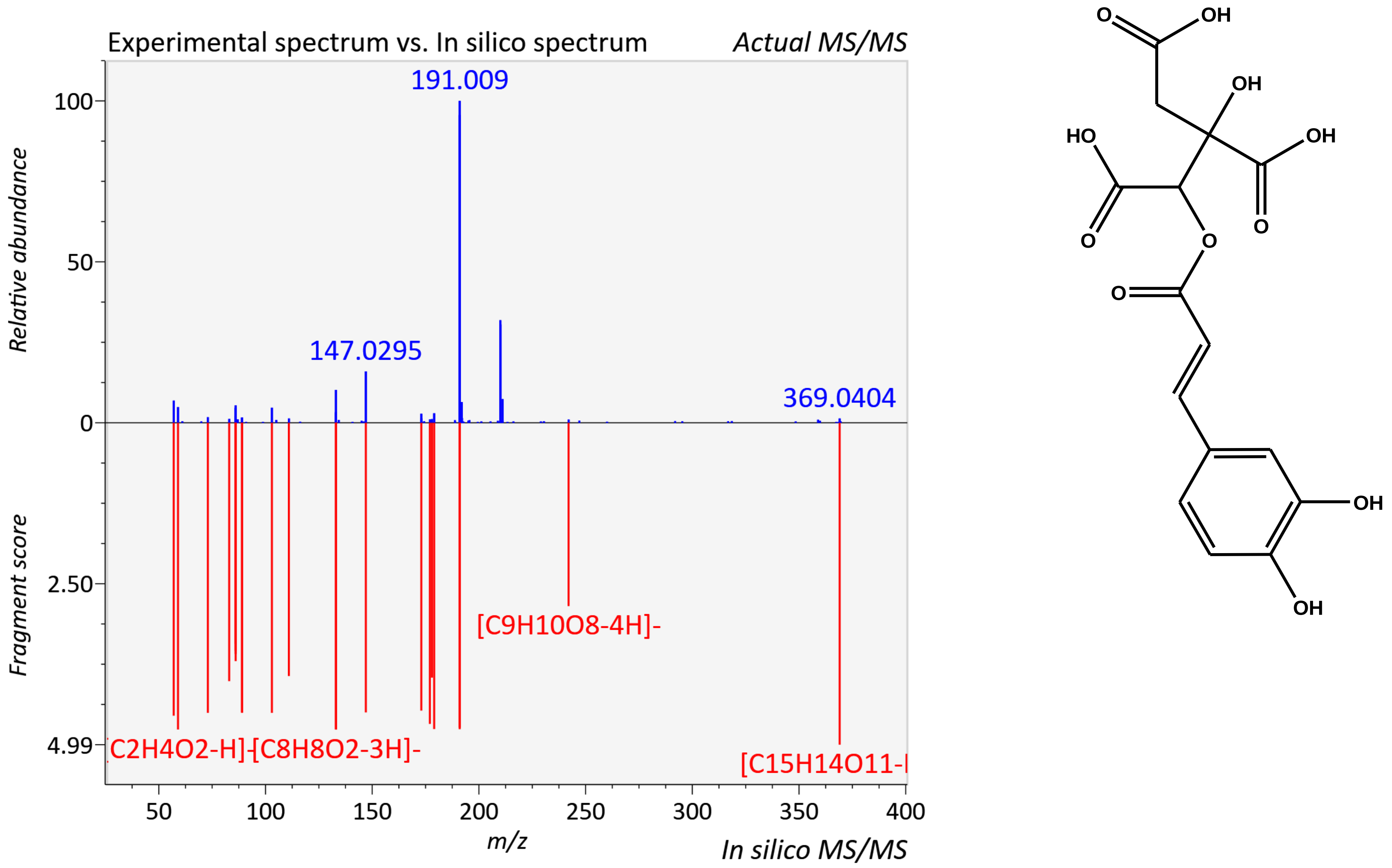

Supplementary Figure….MS/MS spectra for 2-O-caffeoylhydroxycitric acid

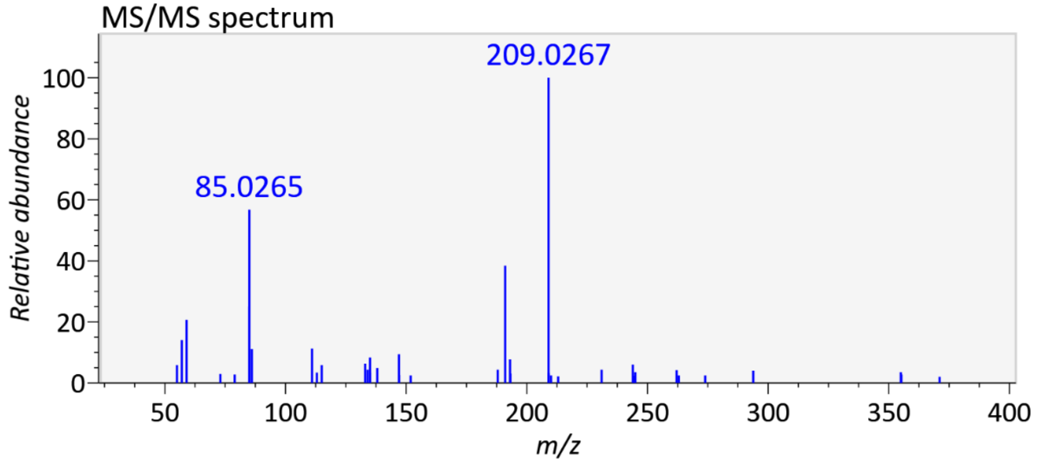

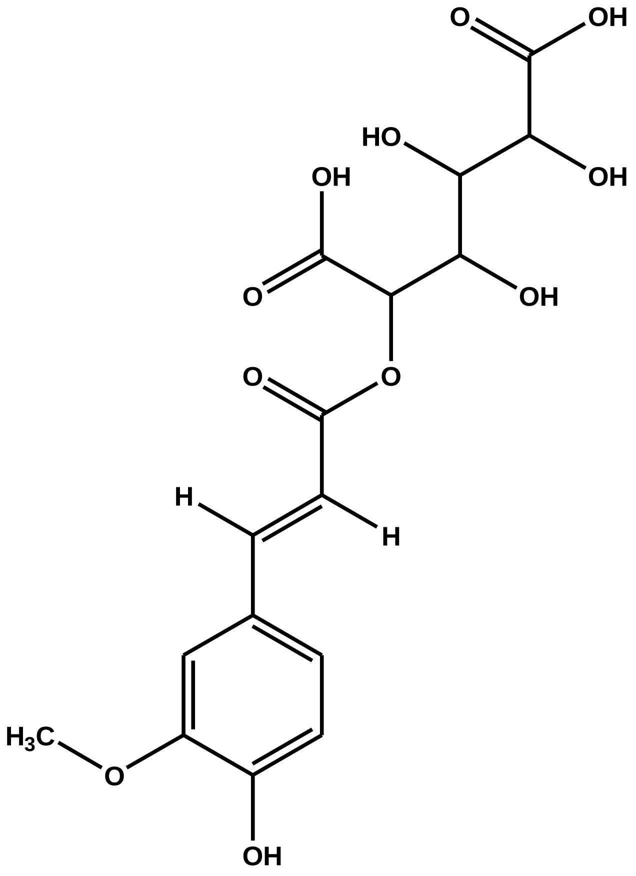

Supplementary Figure… MS/MS spectra for 2-(E)-O-feruloyl-D-galactaric acid

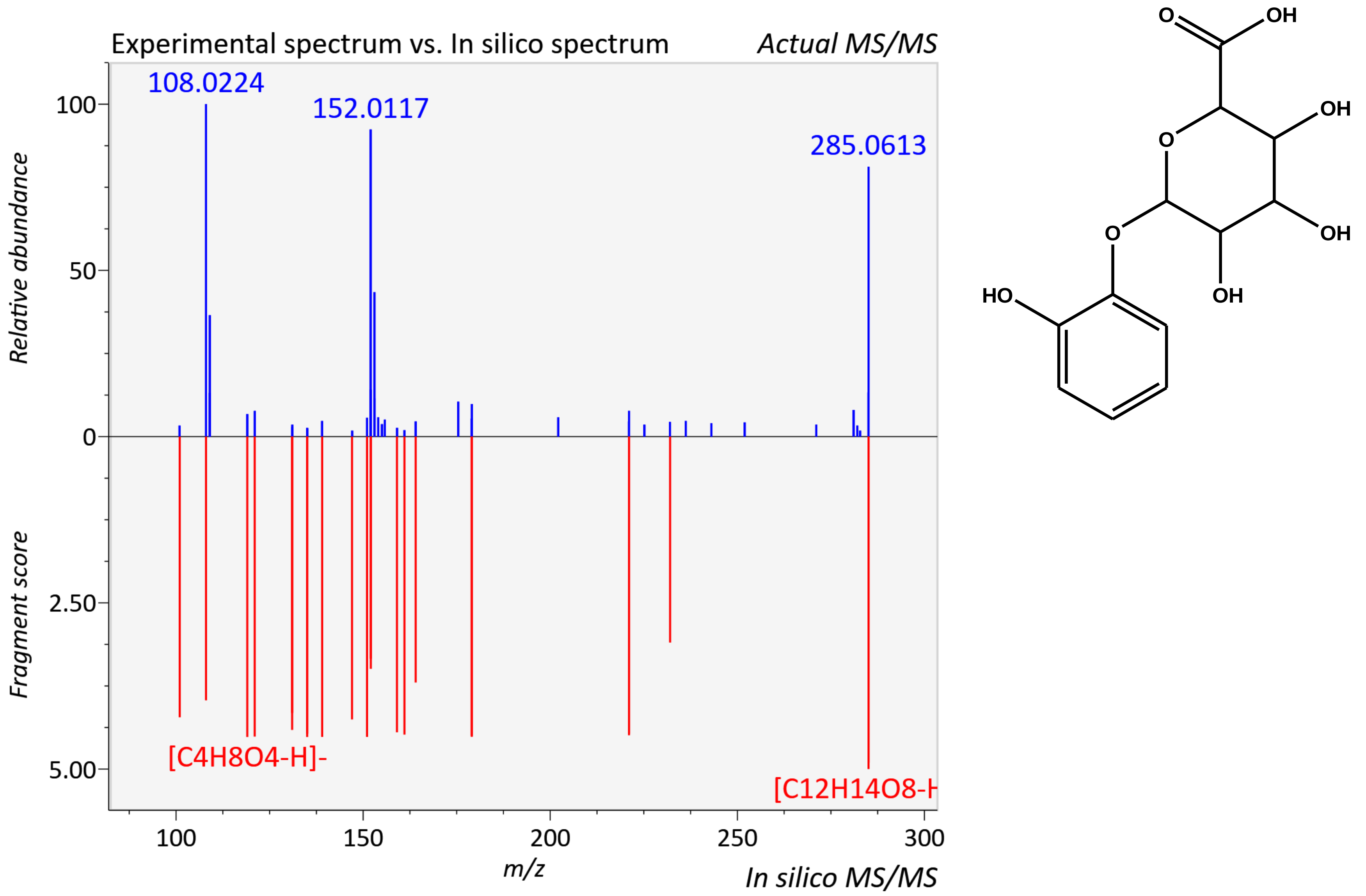

Supplementary Figure..MS/MS spectra for Diphenol glucuronide

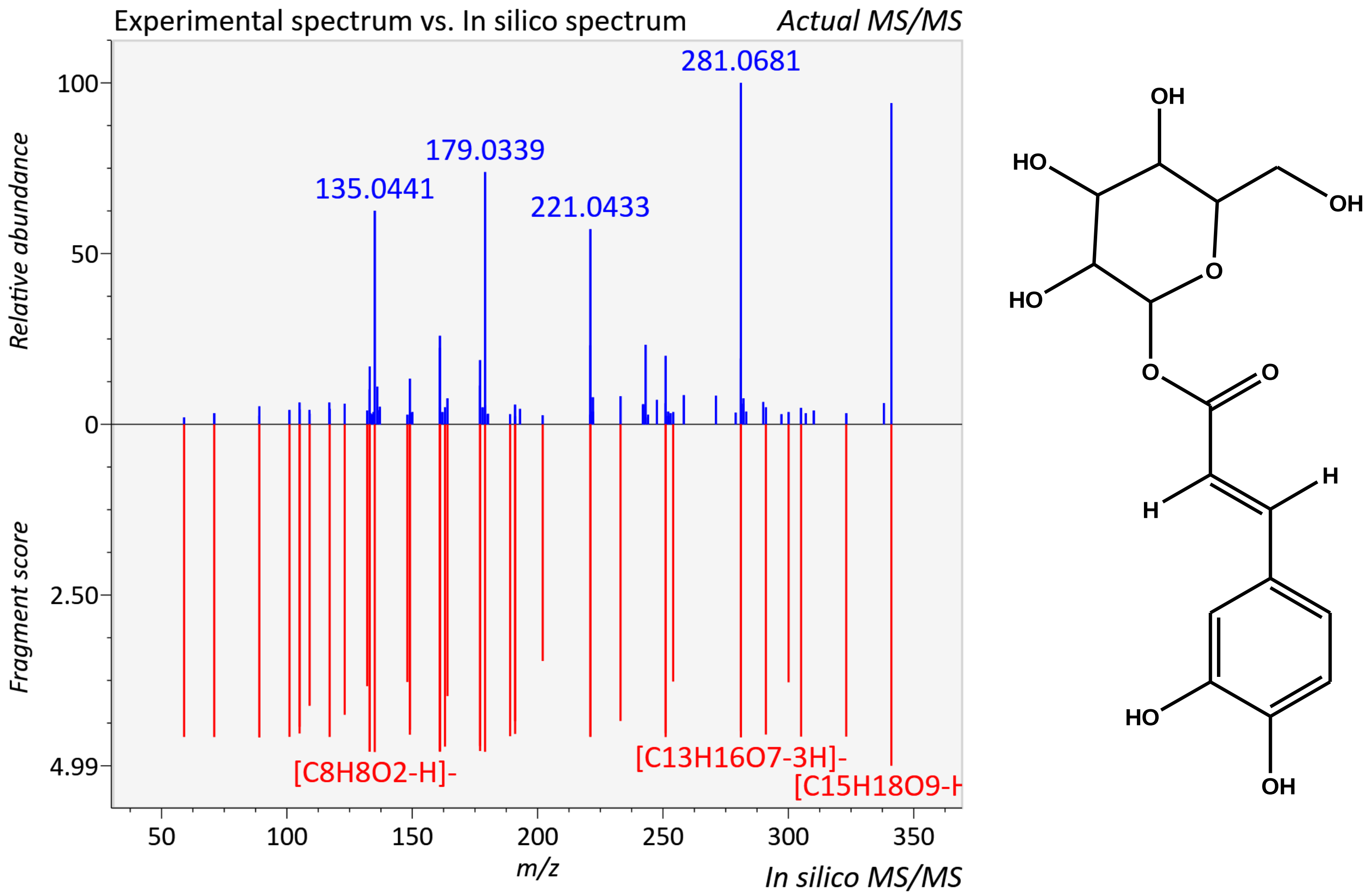

Supplementary Figure…MS/MS spectra for 1-O-caffeoylglucose

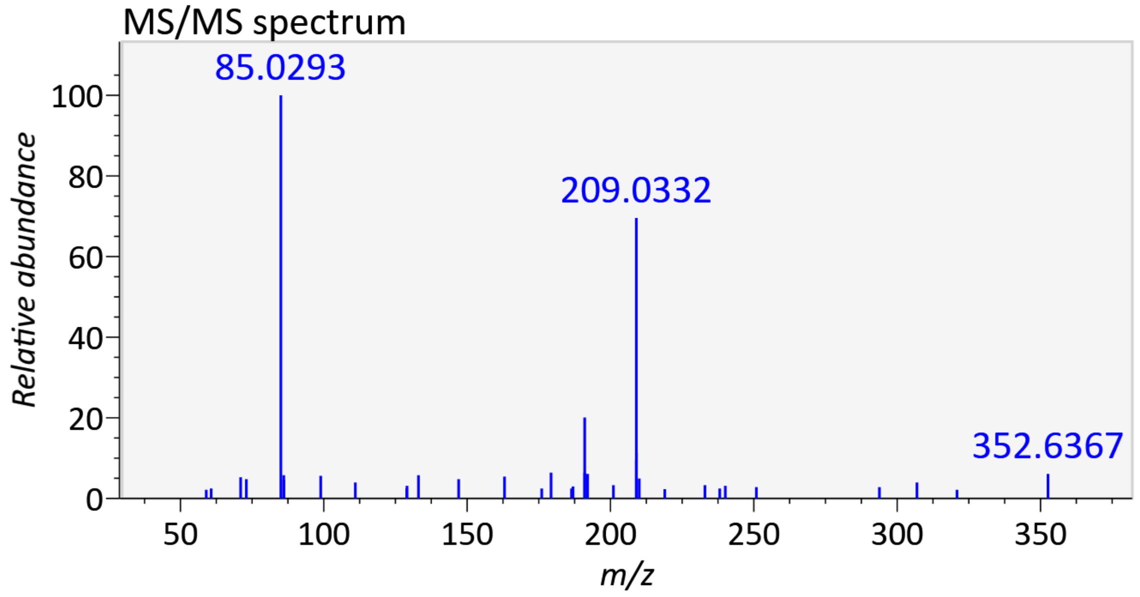

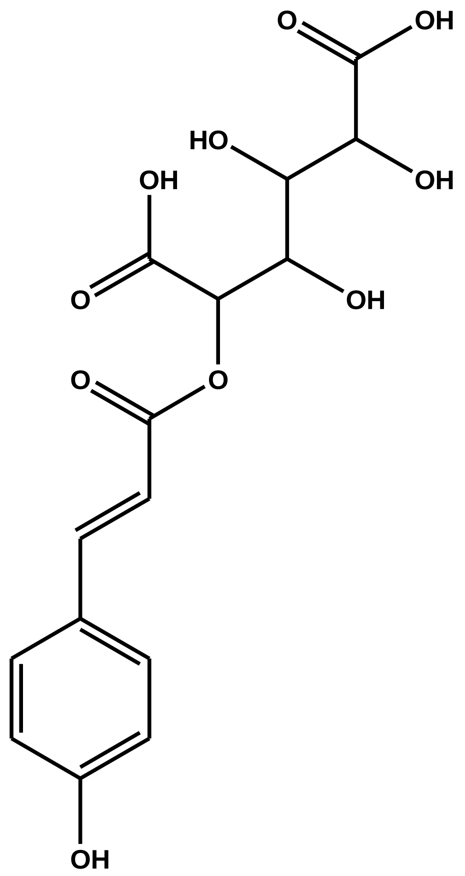

Supplementary Figure… MS/MS spectrum for Coumaroyl glucaric acid

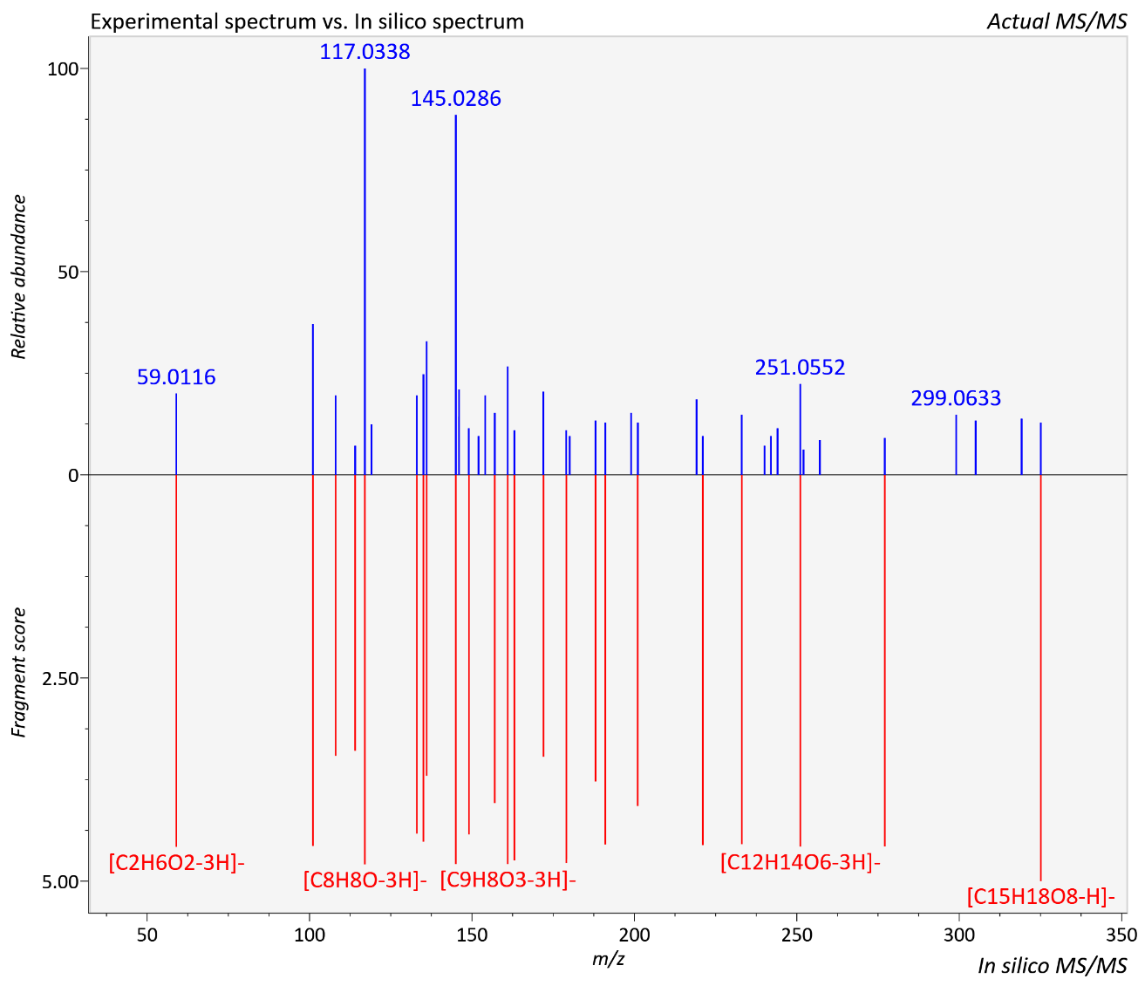

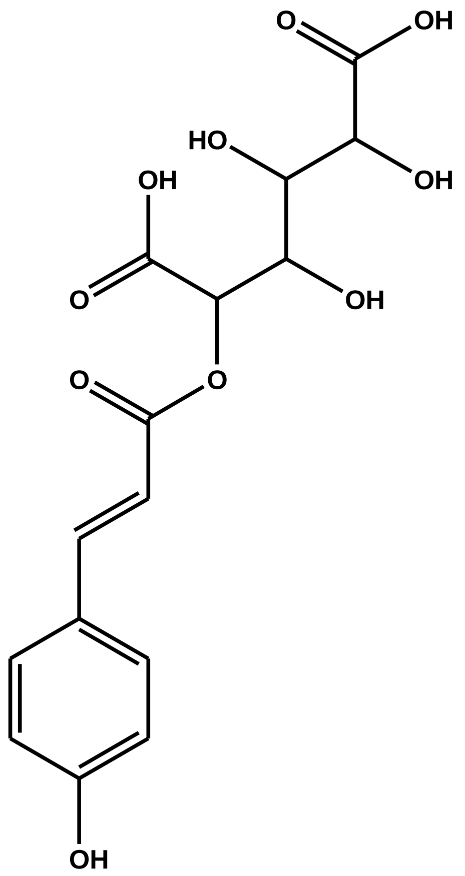

Supplementary Figure….MS/MS spectrum for 1-O-p-Coumaroyl beta D-glucose

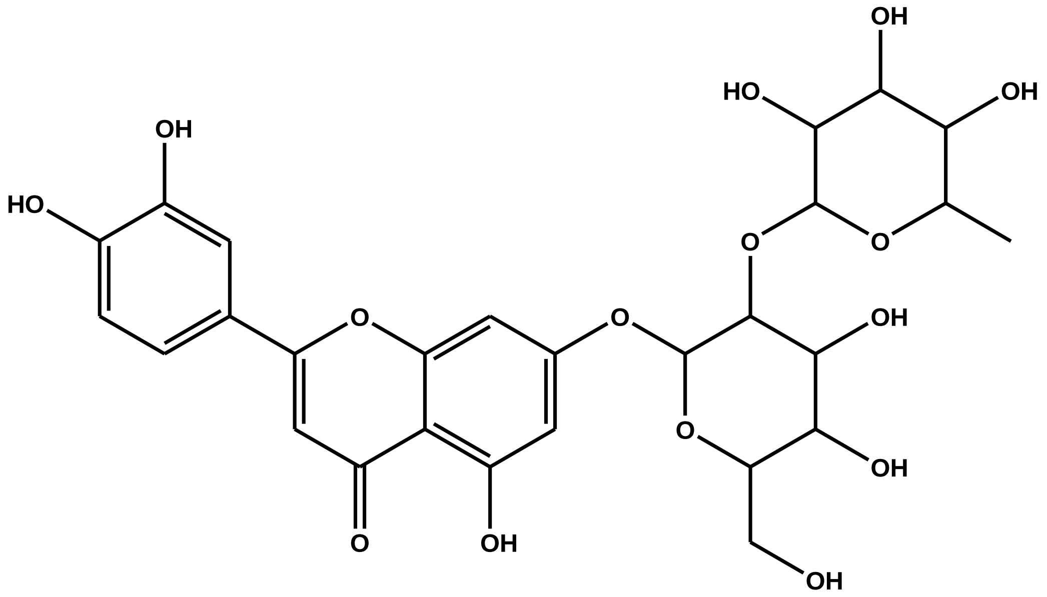

Supplementary Figure…. MS/MS spectrum for Luteolin 7 neohesperidoside

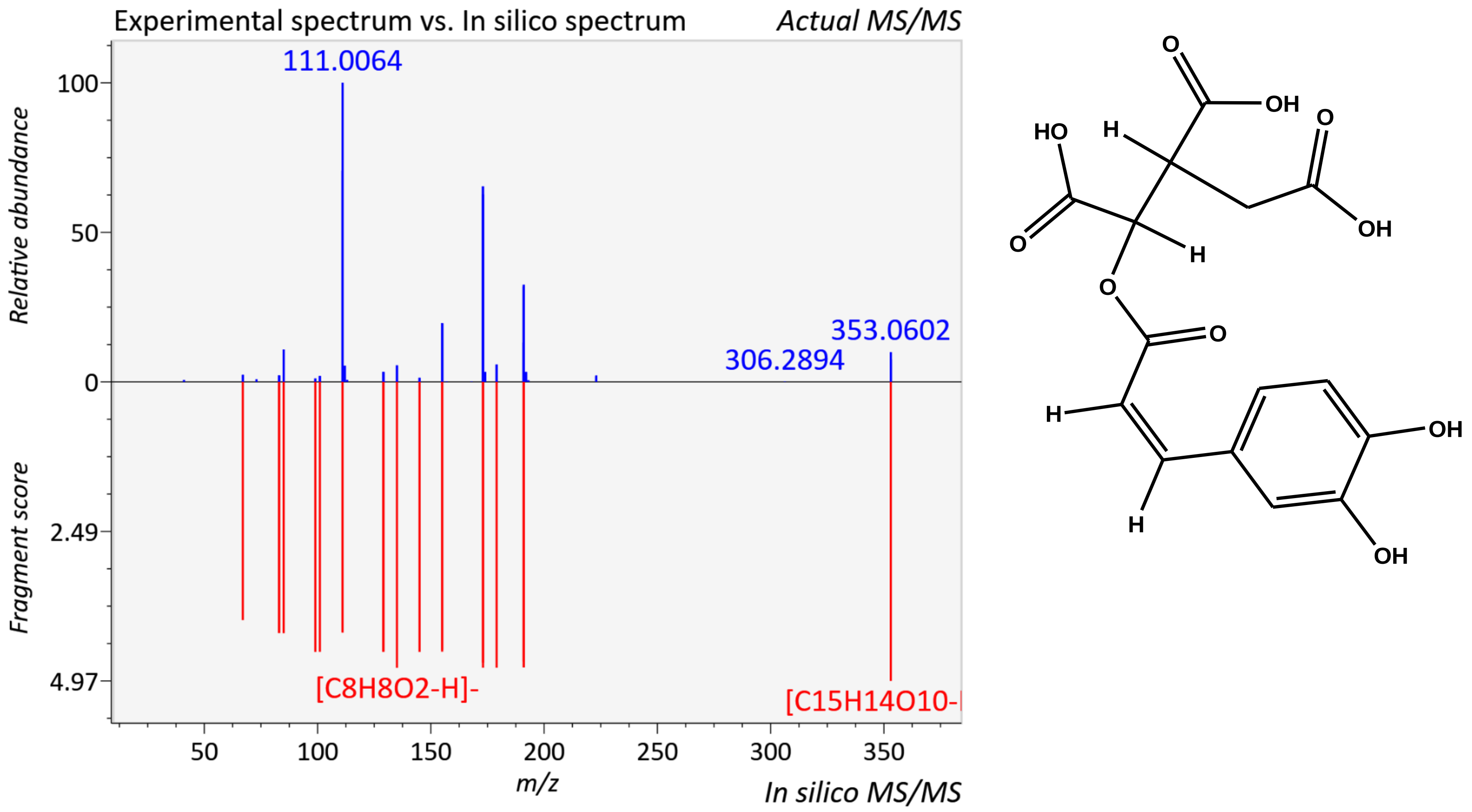

Supplementary Figure…MS/MS spectrum for 2 caffeoylisocitric acid

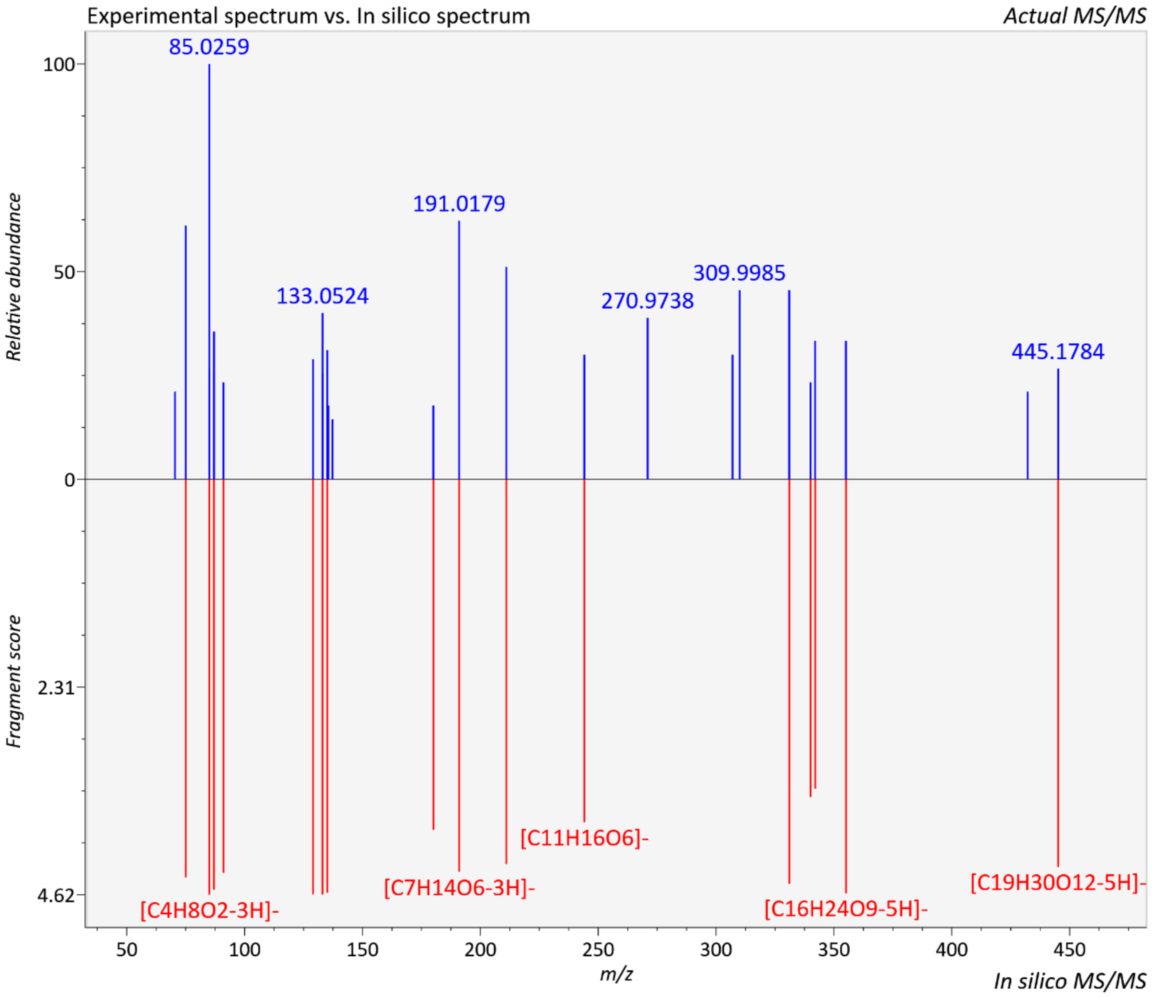

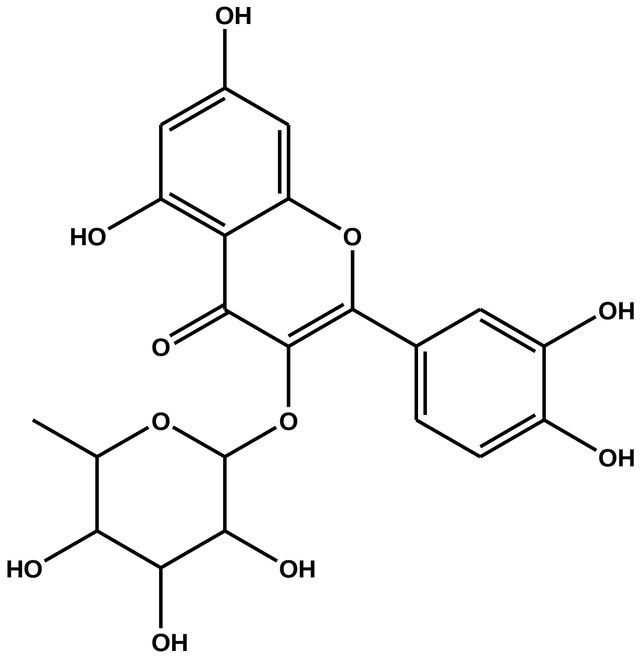

Supplementary Figure…MS/MS spectrum for quercetrin

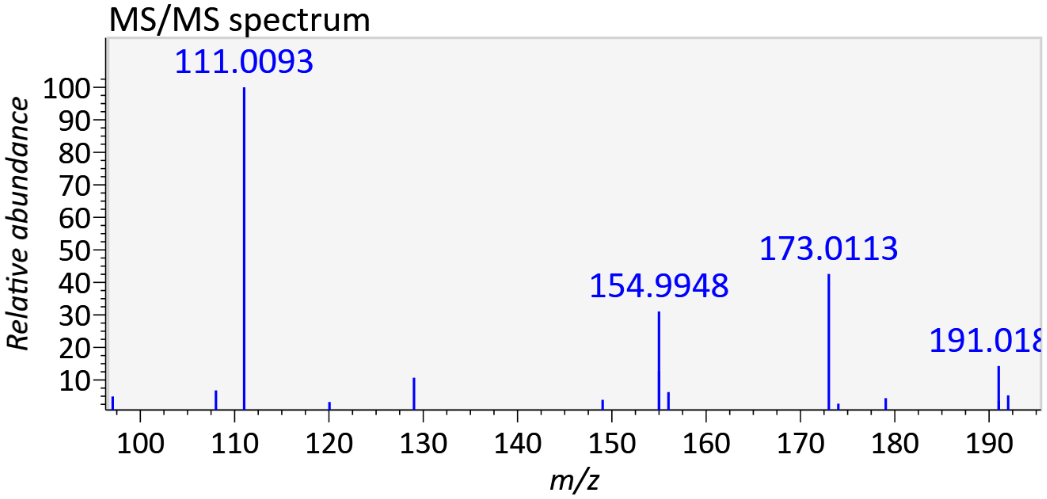
MS/MS spectrum
111.0093
154.9948
173.0113
191.018
Relative abundance
10
20
30
40
50
60
70
80
90
100
100
110
120
130
140
150
160
170
180
190
m/z

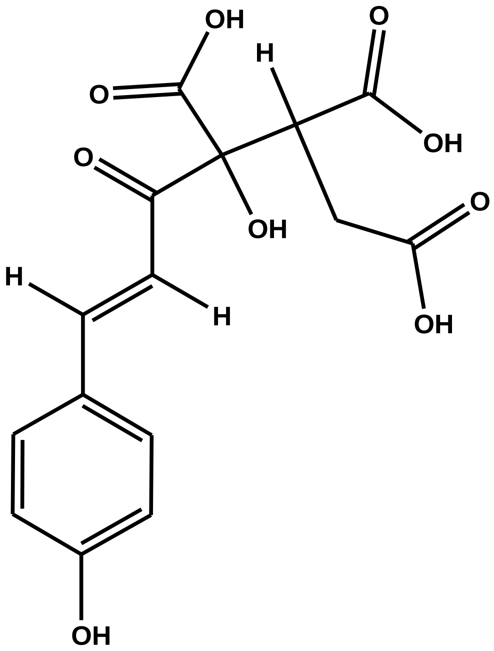
OH
O
H
O
OH
O
OH
O
H
H
OH
OH

Supplementary Figure… MS/MS spectrum for coumaroyl isocitrate

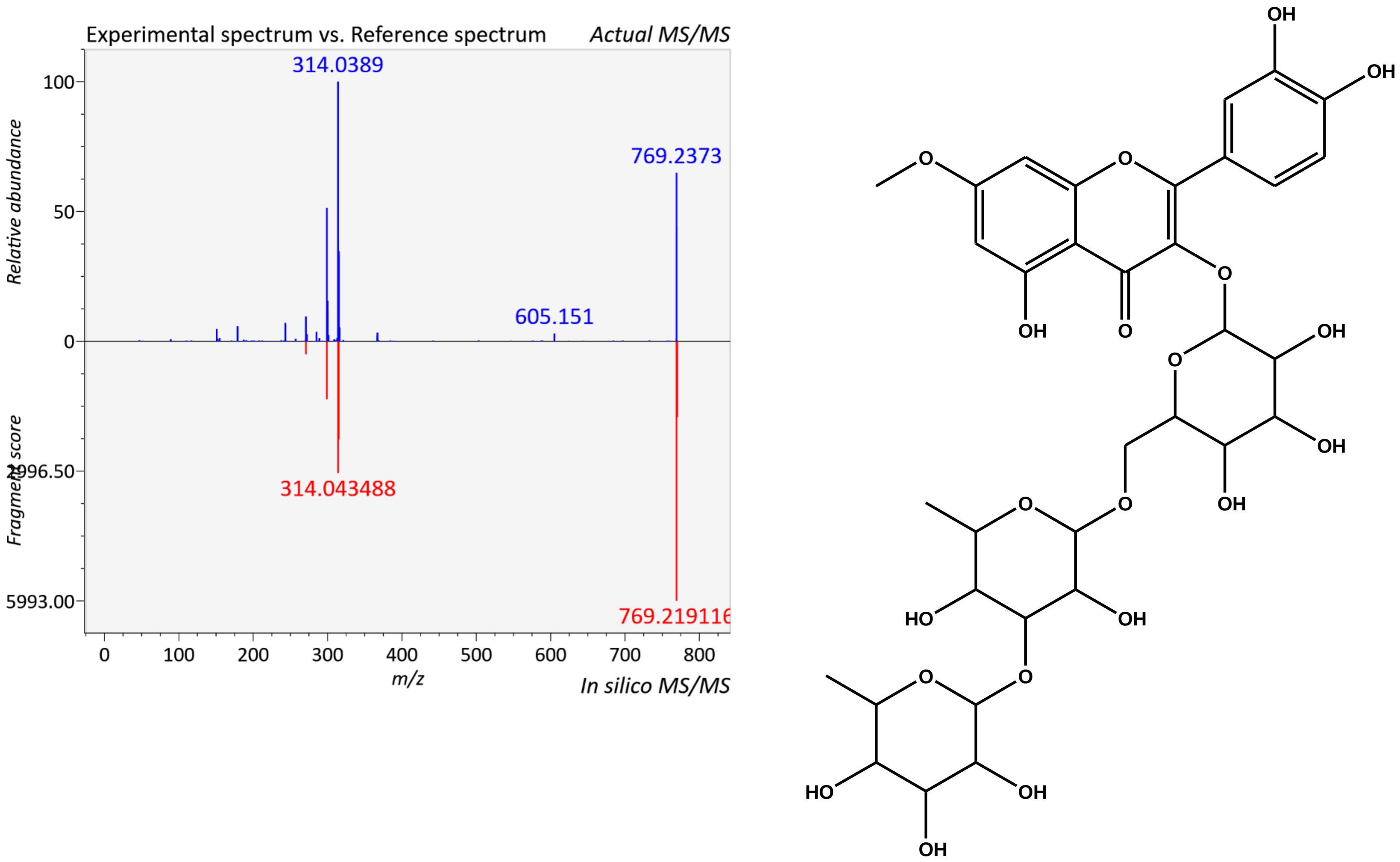

Supplementary Figure….MS/MS spectrum for 7-Methylquercetin-3-Galactoside-6''-Rhamnoside-3'''-Rhamnoside

Supplementary Figure…MS/MS spectrum for feruloyl isocitrate

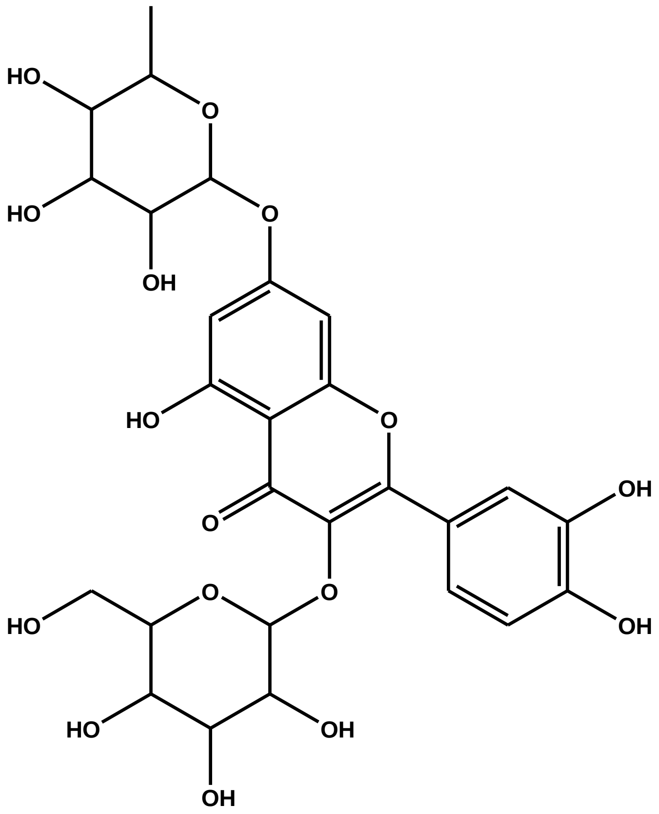

Supplementary Figure…MS/MS spectrum for Quercetin 3-galactoside 7-rhamnoside

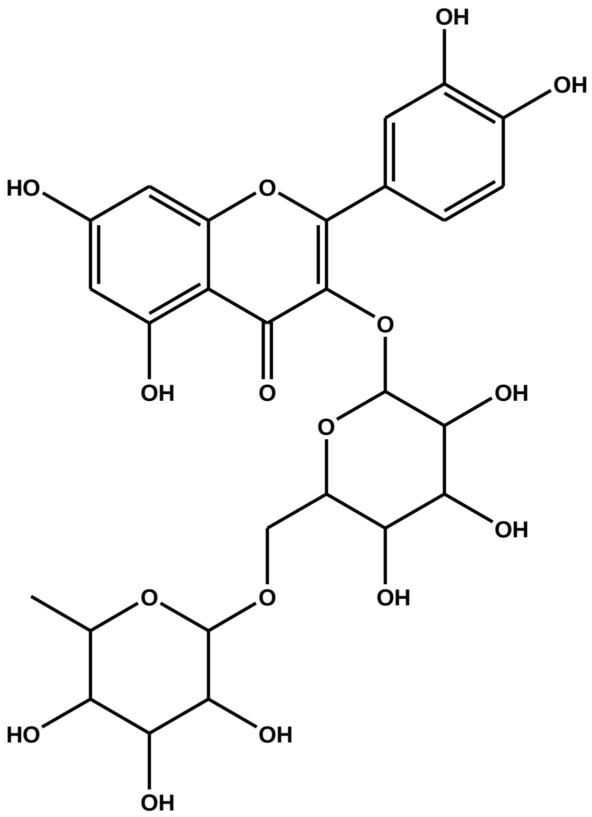

Supplementary Figure….MS/MS spectrum for Rutin

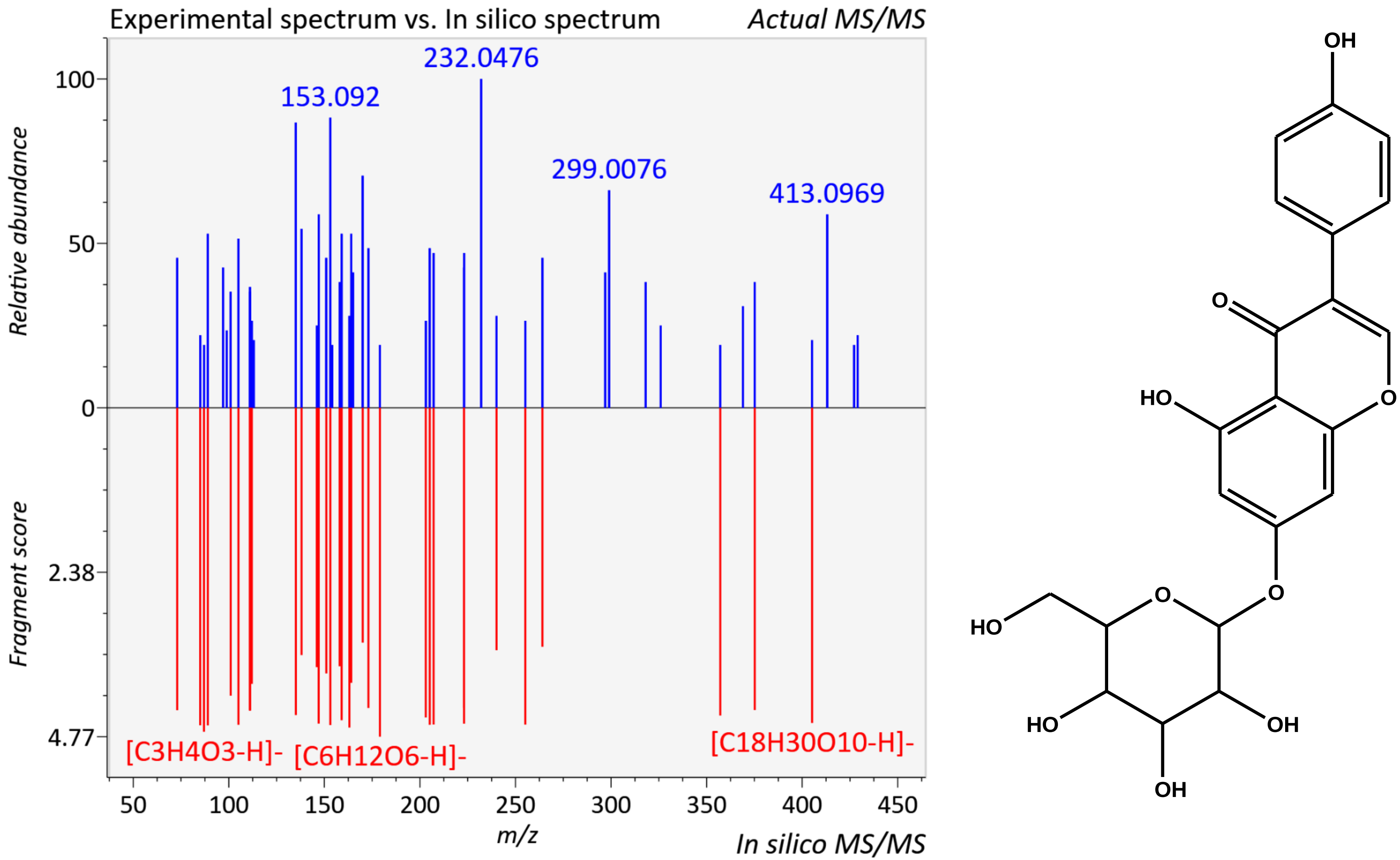

Supplementary Figure….MS/MS spectrum for genistin

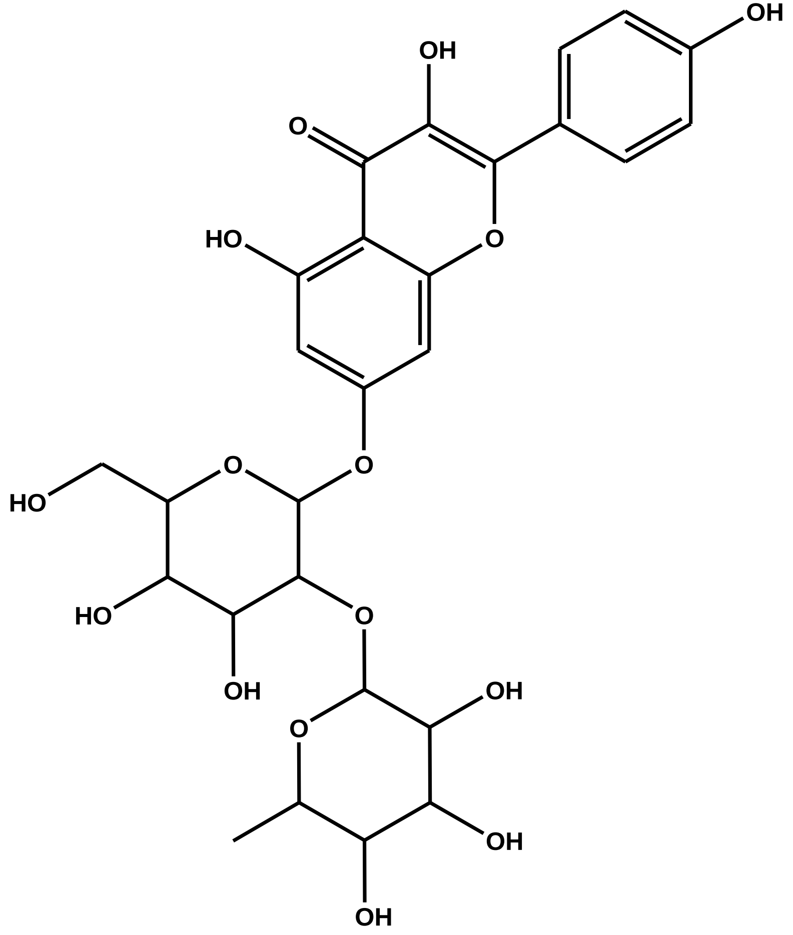

Supplementary Figure….MS/MS spectrum for kaempferol 7-neohesperidoside

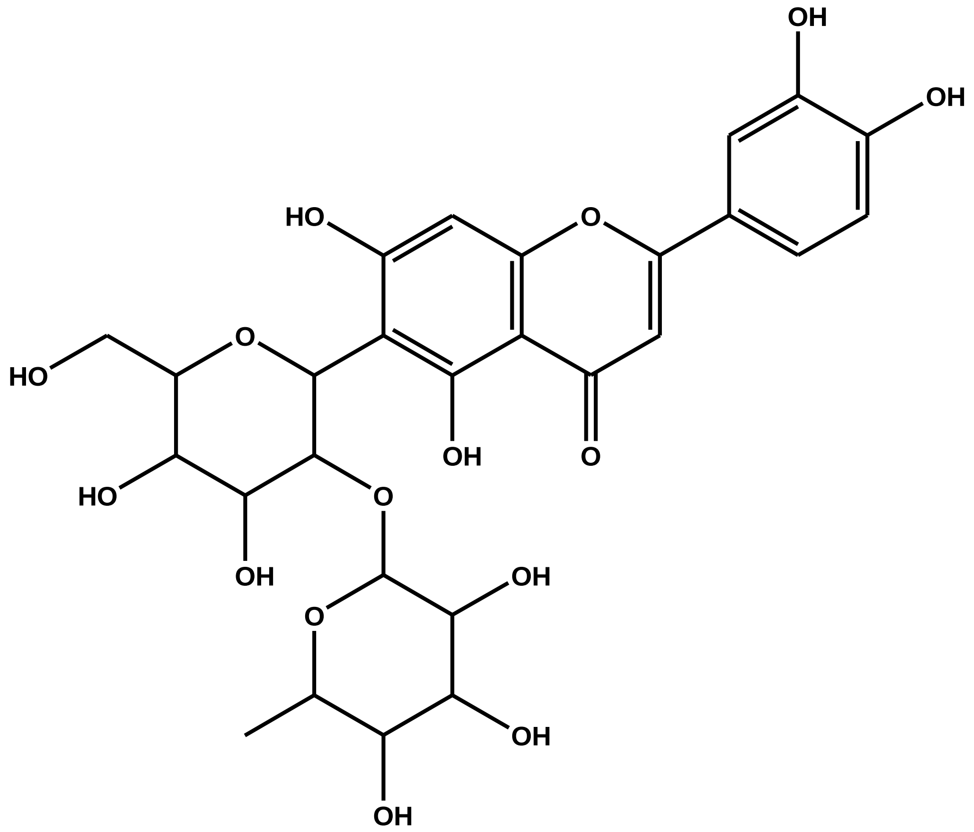

Supplementary Figure……MS/MS spectrum for Isoorientin 2''-O-rhamnoside

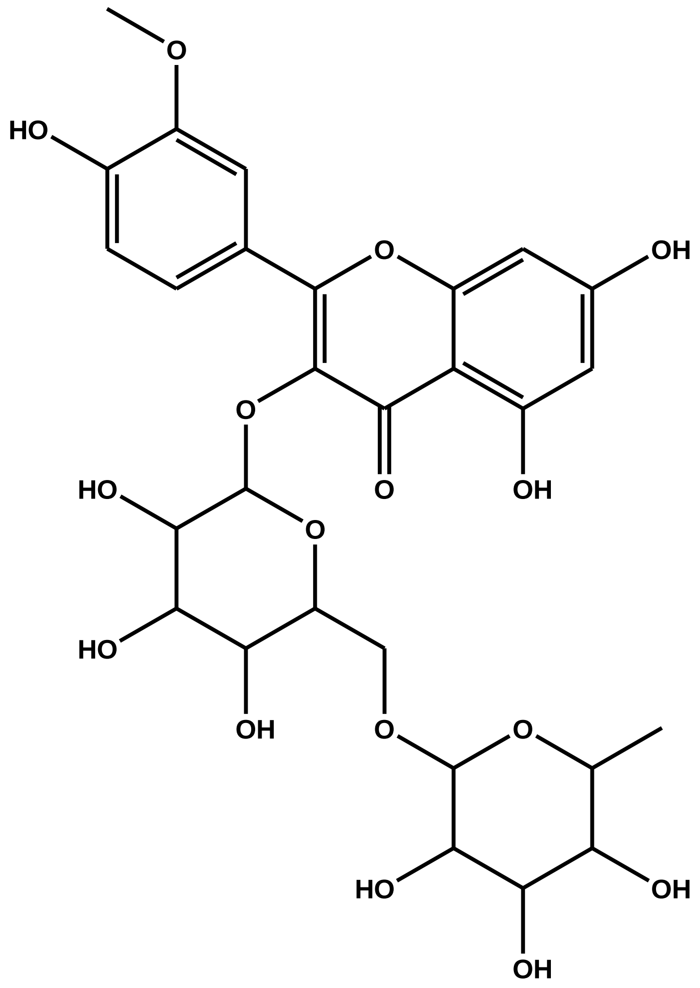

Supplementary Figure…MS/MS spectrum for Isorhamnetin-3-Galactoside-6"-Rhamnoside

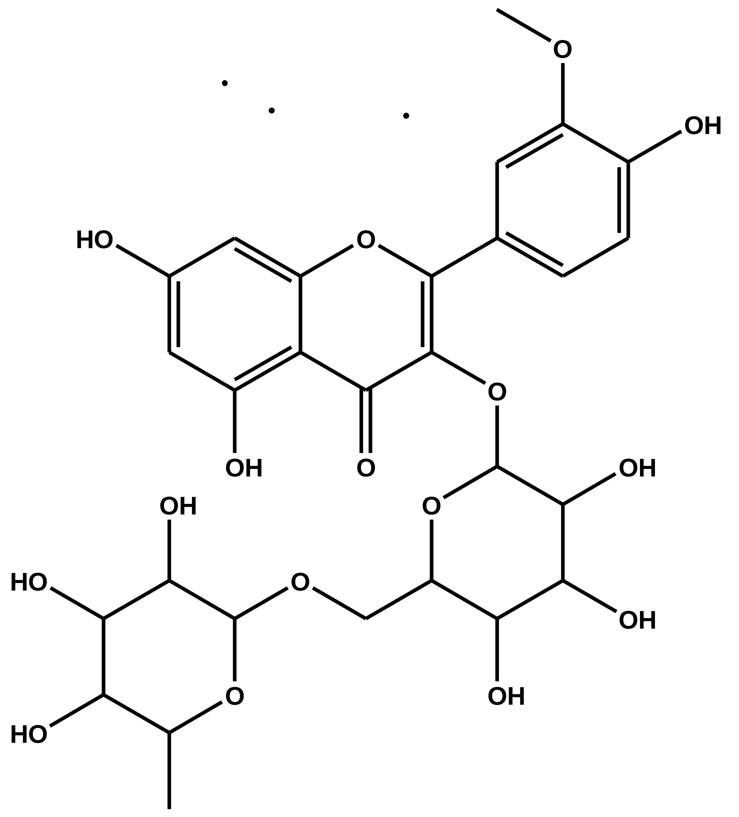

Supplementary Figure….MS/MS spectrum for Isorhamnetin-3-O-rutinoside

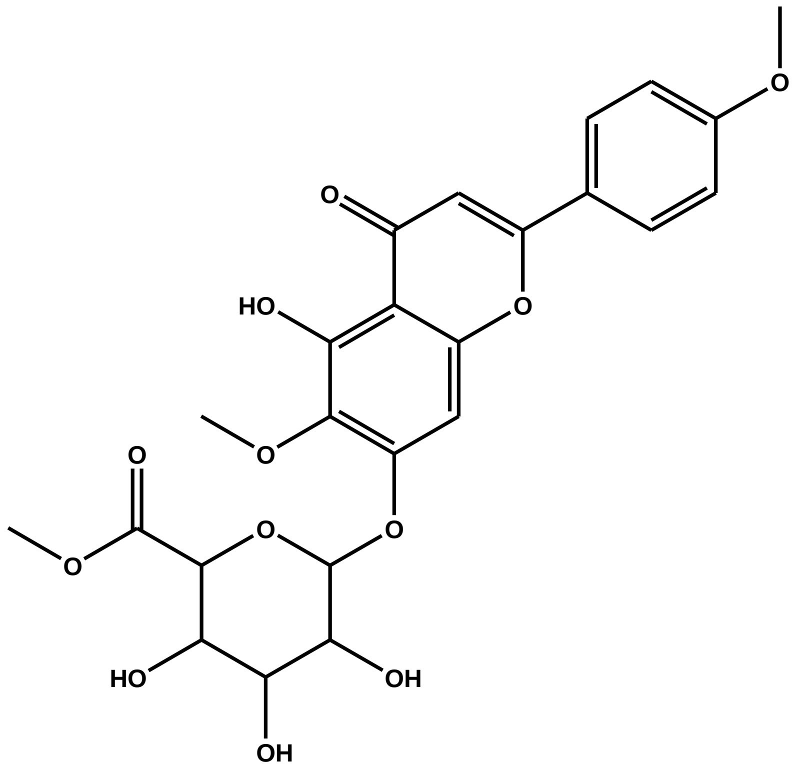

Supplementary Figure…..MS/MS spectrum for Pectolinarigenin 7-(6"-methylglucuronide)
