# Supplementary material for: Changes in Phenolic Metabolites and Biological Activities of Pumpkin Leaves (Cucurbita moschata Duchesne ex Poir.) During Blanching
Source: Front Nutr. 2021 Mar 15;8:641939. doi: 10.3389/fnut.2021.641939 (PMC8005549; doi:10.3389/fnut.2021.641939)
Supplement: Supplementary file 2 [file Data_Sheet_2.PDF]

Supplementary Figure 1 Calibration of cocktail standards, LOD and LOQ values

Compound name: catechin
Correlation coefficient: r = 0.999972, r^2 = 0.999945
Calibration curve: 53.2446 * x + 53.3416
Response type: External Std, Area
Curve type: Linear, Origin: Exclude, Weighting: 1/x, Axis trans: None

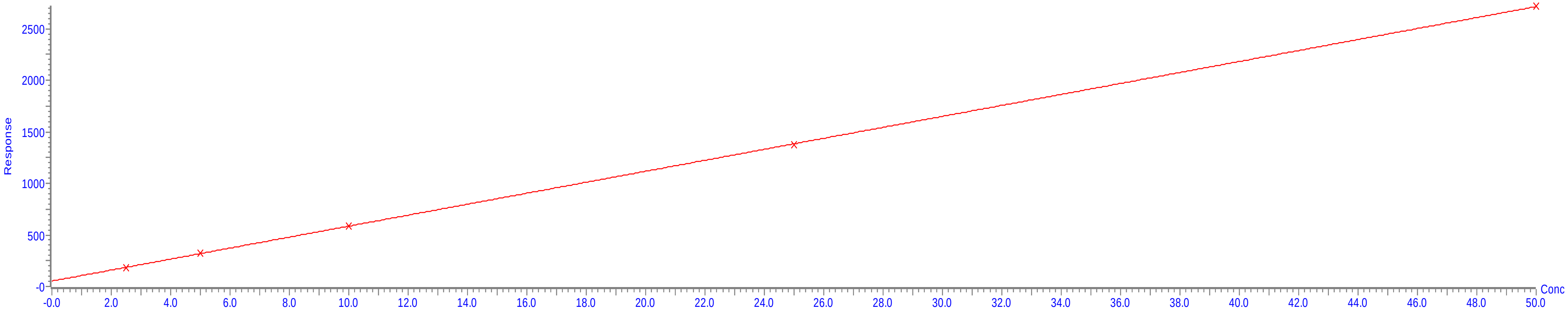

Compound name: epicatechin
Correlation coefficient: r = 0.995703, r^2 = 0.991425
Calibration curve: 59.5242 * x + 103.986
Response type: External Std, Area
Curve type: Linear, Origin: Exclude, Weighting: 1/x, Axis trans: None

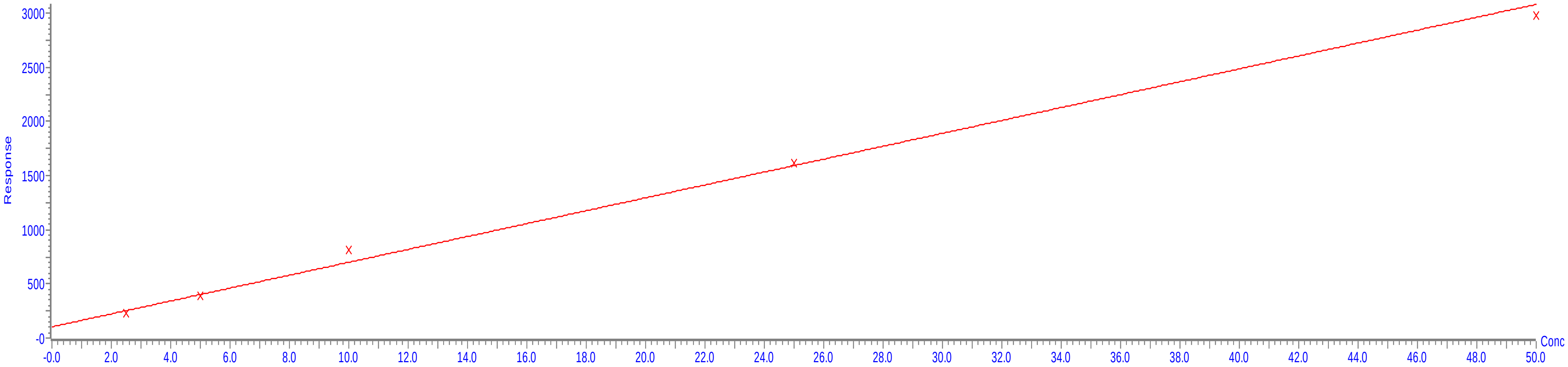

Compound name: Rutin
Correlation coefficient: r = 0.999000, r^2 = 0.998002
Calibration curve: 43.4106 * x + 109.461
Response type: External Std, Area
Curve type: Linear, Origin: Exclude, Weighting: 1/x, Axis trans: None

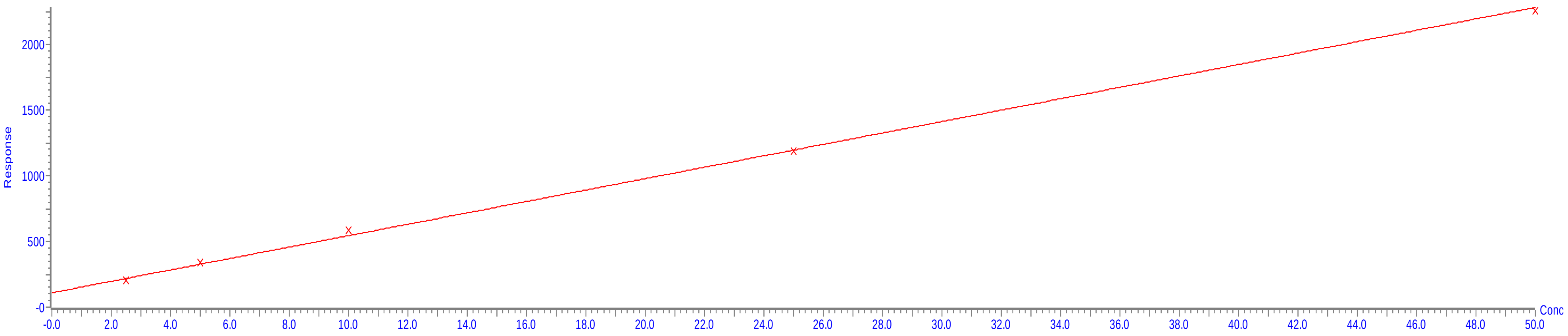

	Name	Sample Text	Catechin Response	Epicatechin Response	Rutin Response
1	DS_TUT_200813_51	Blank	0,269	-	30,84
2	DS_TUT_200813_52	2.5 ppm phenolics	184,811	230,878	206,517
3	DS_TUT_200813_53	5 ppm phenolics	321,852	393,543	334,931
4	DS_TUT_200813_54	10ppm phenolics	590,982	816,574	581,193
5	DS_TUT_200813_55	25 ppm phenolics	1374,29	1611,743	1186,428
6	DS_TUT_200813_56	50 ppm phenolics	2719,897	2973,184	2253,722

Catechin		Epicatechin			RUTIN
conc X	abs Y	ConcX	Abs Y	Conc X	Abs Y
0	0,269	0	0	0	30,84

2,5	184,811	2,5	230,878	2,5	206,517
5	321,852	5	393,543	5	334,931
10	590,982	10	816,574	10	581,193
25	1374,29	25	1611,743	25	1186,428
50	2719,897	50	2973,184	50	2253,722
LOD	3,3*(Sb/a)	LOD	3,3*(Sb/a)	LOD	3,3*(Sb/a)
LOQ	10*(Sb/a)	LOQ	10*(Sb/a)	LOQ	10*(Sb/a)
Steyx (Sb)	23,01649	Sb	90,23955	Sb	43,37247
Slope (a)	53,70335	a	58,33423	a	43,45472
LOD	1,414333	LOD	5,104902	LOD	3,293753
LOQ	4,285857	LOQ	15,4694	LOQ	9,98107
